# International survey on invasive lobular breast cancer identifies priority research questions

**DOI:** 10.1038/s41523-024-00661-3

**Published:** 2024-07-20

**Authors:** Steffi Oesterreich, Leigh Pate, Adrian V. Lee, Fangyuan Chen, Rachel C. Jankowitz, Rita Mukhtar, Otto Metzger, Matthew J. Sikora, Christopher I. Li, Christos Sotiriou, Osama S. Shah, Thijs Koorman, Gary Ulaner, Jorge S. Reis-Filho, Nancy M. Davidson, Karen Van Baelen, Laurie Hutcheson, Siobhan Freeney, Flora Migyanka, Claire Turner, Patrick Derksen, Todd Bear, Christine Desmedt

**Affiliations:** 1 University of Pittsburgh, Department of Pharmacology and Chemical Biology, UPMC Hillman Cancer Center, Women’s Cancer Research Center, Magee Womens Research Institute, Pittsburgh, PA USA; 2 Independent ILC Advocate, Founder LBCA, Pittsburgh, PA USA; 3 Institute for Precision Medicine, University of Pittsburgh and UPMC, Pittsburgh, PA USA; 4 Tsinghua University, Beijing, China; 5 University of Pennsylvania and Penn Medicine Abramson Cancer Center, Philadelphia, PA USA; 6 University of California, San Francisco, Department of Surgery, San Francisco, CA USA; 7 Dana Farber Cancer Institute, Department of Medical Oncology, Boston, MA USA; 8 Dept. of Pathology, University of Colorado Anschutz Medical Campus, Aurora, CO USA; 9 Fred Hutchinson Cancer Center, Division of Public Health Sciences, Seattle, WA USA; 10 Jules Bordet Institute Belgium, Brussels, Belgium; 11 Department of Pathology, University Medical Center Utrecht, Utrecht, The Netherlands; 12 Molecular Imaging and Therapy, Hoag Family Cancer Institute, Molecular Imaging and Therapy, Irvine, CA. Departments of Radiology and Translational Genomics, University of Southern California, Los Angeles, CA USA; 13 Memorial Sloan Kettering Cancer Center, New York, NY USA; 14 Laboratory for Translational Breast Cancer Research, Department of Oncology, KU Leuven, Leuven, Belgium; 15 Lobular Breast Cancer Alliance Inc., White Horse Beach, MA USA; 16 Lobular Ireland, Dublin, Ireland; 17 Dynami Foundation, Wausau, WI USA; 18 Lobular Breast Cancer UK, Nottingham, UK; 19 Department of Family Medicine, University of Pittsburgh, Pittsburgh, patients/advocates, Pittsburgh, PA USA; 20 Present Address: AstraZeneca, GAITHERSBURG, MARYLAND, USA

**Keywords:** Oncology, Breast cancer

## Abstract

There is growing awareness of the unique etiology, biology, and clinical presentation of invasive lobular breast cancer (ILC), but additional research is needed to ensure translation of findings into management and treatment guidelines. We conducted a survey with input from breast cancer physicians, laboratory-based researchers, and patients to analyze the current understanding of ILC, and identify consensus research questions. 1774 participants from 66 countries respondents self-identified as clinicians (*N* = 413), researchers (*N* = 376), and breast cancer patients and advocates (*N* = 1120), with some belonging to more than one category. The majority of physicians reported being very/extremely (41%) to moderately (42%) confident in describing the differences between ILC and invasive breast cancer of no special type (NST). Knowledge of histology was seen as important (73%) and as affecting treatment decisions (51%), and most agreed that refining treatment guidelines would be valuable (76%). 85% of clinicians have never powered a clinical trial to allow subset analysis for histological subtypes, but the majority would consider it, and would participate in an ILC clinical trials consortium. The majority of laboratory researchers, reported being and very/extremely (48%) to moderately (29%) confident in describing differences between ILC and NST. They reported that ILCs are inadequately presented in large genomic data sets, and that ILC models are insufficient. The majority have adequate access to tissue or blood from patients with ILC. The majority of patients and advocates (52%) thought that their health care providers did not sufficiently explain the unique features of ILC. They identified improvement of ILC screening/early detection, and identification of better imaging tools as top research priorities. In contrast, both researchers and clinicians identified understanding of endocrine resistance and identifying novel drugs that can be tested in clinical trials as top research priority. In summary, we have gathered information from an international community of physicians, researchers, and patients/advocates that we expect will lay the foundation for a community-informed collaborative research agenda, with the goal of improving management and personalizing treatment for patients with ILC.

## Introduction

Breast cancer is the most frequently diagnosed cancer with one in eight woman being diagnosed in their lifetime^1^. Invasive breast cancers exhibit different histological subtypes with the majority (~75–80%) being carcinoma of no special type (NST), previously referred to as invasive ductal carcinoma (IDC). Invasive lobular breast cancer (ILC) is the most common special type, representing 10–15% of all breast cancers^2^. The hallmark of ILC is loss of E-cadherin, resulting in discohesive cells and alteration of tumor cellular morphology. These are the foundation for the histopathological diagnosis^3^. The diagnosis of ILC remains challenging in part due to the existence of different ILC subtypes and a lack of consistency of diagnostic methodology^4^. Recent studies have shown that interobserver agreement in diagnosis of ILC can be increased with the use of E-cadherin immunohistochemistry (IHC) as a diagnostic marker^5^, and although efforts to improve this have recently been published (https://pubmed.ncbi.nlm.nih.gov/38641322/), at this point in time there are no guidelines recommending its use. The pathognomonic feature of ILC is genetic inactivation of *CDH1*^6^. Compared to patients with NST, patients with ILC are older, and at the time of diagnosis tumors are mostly estrogen receptor-positive, larger, and of higher stage, likely due to limitations of imaging modalities for detection of lobular tumors^7^. Patients with ILC suffer more frequently from late recurrences (often to less common sites such as the gastrointestinal and urogenital tract) than patients with NST, resulting in worse long-term outcome despite fewer high-risk patients being identified by molecular profiling^8^. There are limited studies comparing response to chemotherapy, but collectively they suggest decreased efficacy in patients with ILC^9,10^. Despite the increasing realization of unique biology, etiology, and progression of ILC, ILC is understudied relative to other breast cancers, and there are no treatment guidelines specifically for ILC^7^, however some clinical trials specific for or enriching for patients with ILC are currently being performed.

Here we undertook a worldwide survey, which included the three major stakeholders – breast cancer clinicians/researchers, laboratory-based researchers, and the community including patients and patient advocates. Using this survey, we analyzed the current understanding of ILC and identified consensus research questions on ILC, which can provide the foundation for future collaborative studies.

## Results

### Demographics and characteristics of survey respondents

In total, 1714 individuals were contacted by email with a 39% response rate and for these responses there was a 95% completion rate in taking the survey. Subsequent additional targeted recruitment and outreach via social media especially Twitter (now X) and Facebook resulted in an additional 1,166 respondents starting the survey. In total, 1774 participants answered at least one question and 1310 finished the survey. Of the survey respondents, 688 asked to be included as having contributed to the survey, and they are listed (Supplementary Data File 1).

**Fig. 1:**
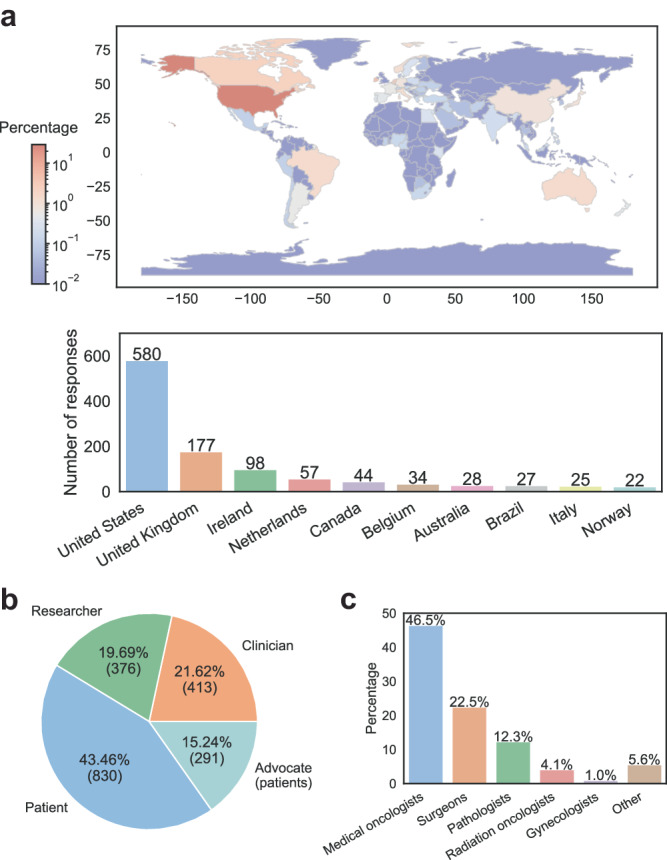
Location of respondents to ILC Survey. **A** A world map showing the percentage by country of respondents to the survey, and the top ten countries with numbers of respondents. World map was generated using https://github.com/geopandas/geopandas. **B** Overall number of physicians, lab- based researchers, and breast cancer patients, with advocates being indicated. **C** Physicians are separated by clinical specialty.

Respondents resided in 66 countries (Supplementary Data File 2) covering all continents except Antarctica (Fig. 1A). The top three countries based on number of respondents were the US (47.65%), UK (9.66%) and Ireland (6.55%).

Respondents self-identified as breast cancer physicians/researchers (*N* = 413), as basic (laboratory-based) researchers (*N* = 376), and breast cancer patients (*N* = 1121) of whom 288 (26.1%) indicated membership in advocacy groups (Fig. 1B). Some respondents belonged to more than one category - 28 clinicians and 17 laboratory-based researchers were also breast cancer patients. There was also overlap between the breast cancer clinicians/researchers and the laboratory-based researcher, with 195 (47.2%) identifying as clinicians with a research lab.

The majority of the breast cancer physicians were medical oncologists (46.5%) and surgeons (22.5%) followed by representation from pathology (12.3%), radiation oncology (4.1%), gynecology (1.0%) and others (5.6%), which included palliative care, patient navigators, clinical oncology, geneticist, family medicine, nuclear medicine and more (Fig. 1C). Most of the clinicians practice in academia (60.6%), followed by private practice (17.4%), and governmental institutions (16.9%). There was an equal distribution of experience: 1–10 years (24.0%), 11–20 years (32.6%), 21–30 years 22.7%), and 31 years and more (19.6%). The majority of physicians treated 11-50 (42.8%) and 51–100 (26.8%) patients per month.

The majority of self-identified laboratory-based researchers work in academic institutions (80.7%), and others in private (9.4%) and governmental (6.7%) institutions. There was a roughly equal distribution of researchers working (50.9%) vs. not working on lobular (49.1%) breast cancer, and 80% of those working on ILC have previously received funding for their work on ILC from a wide range of funders (Supplementary Data File 3). The majority of researchers have been working for 1–10 years (35.7%), followed by 11–20 years (29.7%), 21–30 years (23.7%), 31 years (7.8%) and more (19.6%), and less than 1 year (3.6%).

There were 1121 respondents that indicated that they have or have had breast cancer or in situ carcinoma, called “patients” hence forth. At the time of response to the survey, 62.5% had breast cancer but were currently without evidence of disease, 25.4% were in active treatment for breast cancer, and 19.1% had indicated that their disease had recurred with 8.1% being local and 11.1% being distant recurrences. Lobular breast cancer was the most common histology (63.1%), followed by LCIS (13.6%), DCIS (6.8%), NST (6.3%), mixed ductal/lobular (5.3%) and others/unknown (4.9%). The average age for diagnosis was 52.3 years.

Of the 1121 patients who responded, 288 (26.1%) indicated membership in advocacy groups. Of the total number of respondents to the survey, 403 (23.1% of all respondents) belonged to one or more of 170 different advocacy groups, support groups, and other foundations (Supplementary Data File 4).

### Current understanding of lobular breast cancer by clinicians, and communication with patients

**Fig. 2:**
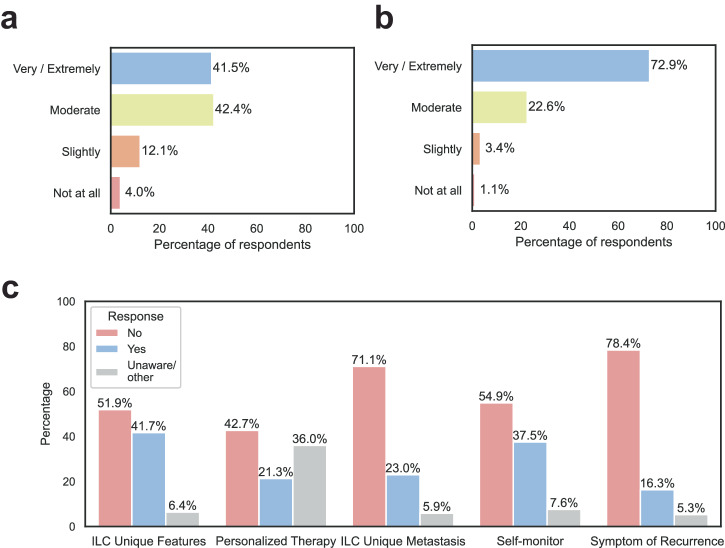
Current understanding of lobular breast cancer by physicians and communication with patients. **A** Responses by clinicians about confidence in describing differences between ILC and NST. **B** Importance of knowledge of histology for physicians. **C** Patients’ responses on communication with physicians. ”Other” refers to “*I was not offered personalized therapy because my physician explained that the treatment is no different for ductal than for lobular*”.

The majority of physicians were confident in describing the differences between ILC and NST (Fig. 2A), and there was no significant difference between medical oncologists, surgeons and other specialties. Only 4% were not all confident, or slightly confident (12%) about the differences. Physicians indicated that knowledge of histology was seen as very/extremely important (73%) (Fig. 2B), and this again was not different between the different specialties The majority of physicians stated that knowledge of histology affected their treatment decisions a lot (51%) or a moderate amount (32%), with surgeons (60%) and others (59%) using histology information significantly more than medical oncologists (40%). Very few physicians (4.5%) felt that knowing histology was not at all or only slightly important. The majority of physicians (57%) indicated that there either was no data, that they did not know, or that they were unsure if there were clinical trials and outcome data supporting unique treatments for ILC. Refining treatment guidelines specifically for lobular breast cancer was seen as valuable for treating patients with ILC in the future by 76% of physicians, for a number of reasons outlined in Supplementary Text File 2.

We asked how patients perceived the knowledge of and specifically, the communication about ILC-specific features by physicians. Many patients (52%) thought that their health care providers did not explain unique features of ILC (Fig. 2C). “Personalized therapy based on histological diagnosis” was discussed with 21% of patients, while it was not discussed with 42%. For 28% of patients, the physicians explained that there was no difference in treatment for NST and ILC. Discussions about potential personalized therapies were held mostly with medical oncologists (89%), followed by surgeons (72%). There were an equal number of radiation oncologists who “definitely did not/probably not” (50%) and who “definitely did/probably did” (50%) discuss ILC-personalized therapy. Most physicians (71%) did not mention that ILC can metastasize to unique places and did not discuss what symptoms, including unusual symptoms, of recurrence the patients should report in the future (78%). For all these communication-related questions, there were differences between countries, with health care providers in the US more frequently being perceived as explaining ILC specific features compared to other countries (Supplementary Data File 5).

Finally, we asked which other ILC-related topics the patients wished they have had a chance to discuss with their physicians. The most common was a discussion of the unique clinical features of ILC, followed by information on recurrence and metastasis, cancer detection and screening, and influence of breast density (Supplementary Data File 6, and Supplementary Fig. 1). All answers were clustered into one of the topics based on semantic similarity, which can be interactively visualized under ‘Topic’ coloring scheme via https://atlas.nomic.ai/data/chelseax488/ilc-survey---discussion-with-doctors/map.

### Current basic/translational research and future priorities identified by survey respondents

For those who self-identified as basic or translational researchers, 48% were very/extremely and 29% were moderately confident in describing differences between ILC and NST. The majority (59.8%) performed none or only a little ILC research with only 20% performing a lot or a great deal of ILC research. Reflecting this, only 23% received funding to work on ILC, and those who focused on ILC were significantly more funded for their ILC work (54%) compared to those who don’t focus on ILC (9%).

There is the need for additional ILC models, as only 11% of respondents found that there were sufficient in vitro and in vivo models for ILC research. 32% of respondents use ILC cell line models in their research, the most common being MDA-MB-134 and SUM44. Majority did not use ILC models due to “lack of facility, resources, or expertise”. 52% of respondents felt that ILC was poorly represented in public genomic datasets, while 69% felt that they were able to obtain lobular breast cancer tissue and/or blood samples from patients with ILC for research.

**Fig. 3:**
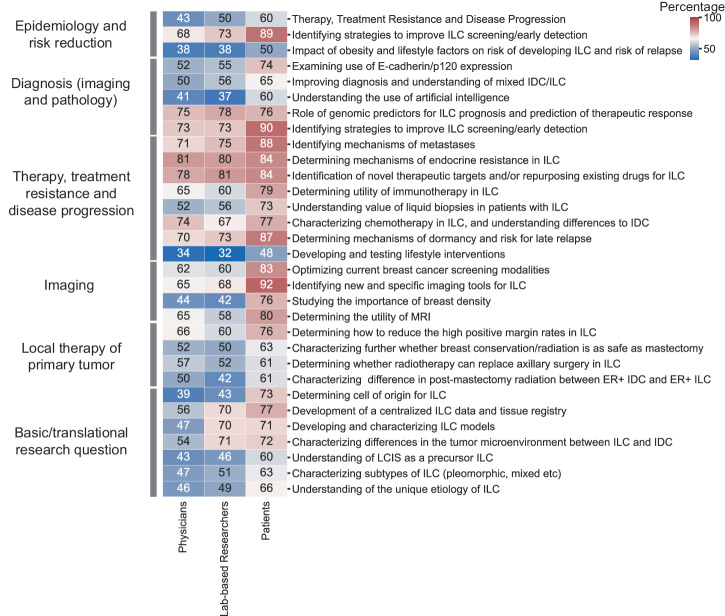
Priority research areas identified by physicians, laboratory researcher, and patients. Heatmap showing percentage of individuals rating each research question as of highest importance (‘most critical and impactful’, against moderate/low importance) among 6 domains of topics in physicians, lab-based researchers, and patients, respectively. Color and number represents the percentage from 0-100 in each block.

We asked the three major stakeholders for their opinions on research priorities in ILC (Fig. 3, and Supplementary Data File 7). We posed questions about 6 major areas with each area having subcategories. The main areas were: (1) Epidemiology and Risk Reduction; (2) Diagnosis (Imaging and Pathologic Analysis), (3) Therapy, treatment resistance and disease progression; (4) Local therapy of the primary tumor; (5) Imaging; and, (6) Lobular tumorigenesis (the formation of tumors), and other basic/translational research questions. There was general agreement in prioritization of research priorities by the physicians and laboratory-based researchers. Both chose “Therapy, treatment resistance and disease progression” as their top research area followed by “Diagnosis (Imaging and Pathology)”. For 5 out of the 6 areas there was agreement on the specific subcategories of interest with the two highest being “Determining mechanisms of endocrine resistance in ILC” and “Understanding value of genomic predictors for ILC prognosis and prediction of therapeutic response”. The largest discordance was in the area of “Basic/translational research” with physicians choosing “Focusing on development of a centralized ILC data and tissue registry” whereas laboratory-based researchers chose “Characterizing differences in the tumor microenvironment between ILC and NST”. The top priority areas for patients were “Imaging” and “Diagnosis (Imaging and Pathology)”. Within “Imaging”, all groups identified “Identifying new and specific imaging tools for ILC” as the key research area, whereas in “Diagnosis (Imaging and Pathologic Analysis)”, patients chose “Identifying strategies to improve ILC screening/early detection” and physicians and researchers choose “Role of genomic predictors for ILC prognosis and prediction of therapeutic response”.

And finally, there was a free text field question asking which other research questions have high priority that might not have been listed. The most frequently mentioned topics (Supplementary Data File 8) among a wide range of answers were: (1) Genetic screening, Germline mutations, Familial risks, (2) Awareness education, (3) New Aromatase Inhibitors (AI) and Selective Estrogen Receptor Degraders (SERDs) and duration of treatment, (4) Genomic predictors/markers, and, (5) Chemotherapy-related questions.

### Clinical trials focused on ILC

Finally, we asked physicians specifically about their opinions on clinical trials in ILC. Half of the physicians reported that “Most of the time/always” approximately half of the clinical trials and studies they were involved in collected data on tumor histology, with twice as many surgeons (35%) than medical oncologists (16%) “always” collecting histology information. Further, 66% of physicians stated that clinical trials they are involved in do not or only sometimes consider histology in their inclusion/exclusion criteria. Most physicians (86%) have not powered clinical trials they were involved in to specifically allow a subset analysis for ILC, and this was not significantly different between surgeons, medical oncologists and other physicians. However, 50% would “probably” and 36% would “definitely” consider powering clinical trials to do subset analysis of lobular breast cancer in the future, for a wide range of reasons (Supplementary Text File 3). Importantly, most physicians (87%) would consider participating in consortia conducting clinical trials in ILC.

## Discussion

ILC is inherently understudied compared to the more common NST subtype of breast cancer. To better understand conceptions about ILC and key research areas, we surveyed patients/advocates, physicians, and laboratory-based researchers. A robust world-wide response (*n* = 1774) was obtained from all three key stakeholder groups. Physicians are confident in describing the differences between ILC and NST and feel that knowledge of histology is important, as it affects their decision making. Importantly, they responded that future refined treatment guidelines would be valuable for patients with ILC. While very few clinical trials have been directed specifically at ILC, most physicians expressed an interest in such trials. Most laboratory researchers felt that ILCs are inadequately presented in large genomic data sets, and there are too few models of the disease. The majority of patients thought that their health care providers did not explain unique features of ILC, and that in general communication was limited. Overall, while there is growing interest in the study and understanding of ILC, there are clear gaps in understanding and presentation of the disease to patients. This challenge is beginning to be addressed by advocacy groups who are developing publicly available educational materials available on websites for examples from the Lobular Beast Cancer Alliance (https://lobularbreastcancer.org/), the European Lobular Breast Cancer Consortium (https://elbcc.org/), and in general there has been an overall increased awareness of unique features of ILC.

Both clinical and laboratory studies are hindered by a lack of studies targeted specifically at ILC and addressing ILC-specific challenges. We asked the three major stakeholders what they felt were the most important areas of ILC research. While there was general concordance between the groups, important areas did show discordance. For example, while physicians and researchers both chose ‘Therapy, treatment resistance and disease progression’ as their top research area, patients/advocates chose ‘Imaging’ and ‘Diagnosis (Imaging and Pathologic Analysis)’. Our survey results are consistent with a recent survey of patients and advocates (*n* = 1476) previously diagnosed with ILC who expressed concerns over current imaging standards^11^. These patients reported that mammography often failed to detect ILC cases until they reach stage 2 or higher, a common issue for ILC. Importantly, tumors were often larger at resection than predicted by imaging. A recent review of the literature also reported that MRI and contrast-enhanced mammogram surpass conventional breast imaging in sensitivity and specificity of ILC detection^12^. There have been recent advances in understanding the reasons for decreased ability to image ILC e.g., reduced uptake of glucose limited FDG-PET^13^ and direct comparisons showing better imaging via FES-PET compared to FDG-PET in ILC^14^. This is clearly an area of patient/advocate interest.

Patients and advocates also highlighted the need for improved diagnosis (imaging and pathologic analysis). This is also consistent with a recent survey of pathologists highlighting the lack of standard definitions for diagnosis of ILC^4^. The current World Health Organization (WHO) classification for ILC diagnosis only requires pathologists to note a non-cohesive growth pattern in the tumor and does not require analysis of E-cadherin status. Highlighting challenges in pathologic diagnosis of ILC, thirty-five pathologists diagnosed NST and ILC from a set of breast cancers and showed only a moderate inter-observer agreement, but a substantial agreement was a found when E-cadherin was also used in the diagnosis^5^. Efforts have recently been done by the pathology working group from the European Lobular Breast Cancer Consortium (ELBCC) to harmonize pathological diagnosis of ILC (https://pubmed.ncbi.nlm.nih.gov/38641322/). Finally, the increased development and use of digital pathology and artificial intelligence, such a recently reported algorithm using 51 different types of clinical and morphological features differentiating between NST and ILC with an AUC of 0.97^15^ may eventually offer the option to improve diagnosis. Another example is a recent study which used a machine learning system to detect ‘*CDH1* biallelic mutations’ as ground truth rather than histology and then developed an AI-based system that can detect ILCs accurately^16^. These algorithms may help resolve the morpho-molecular diagnosis of ILC^17^, particularly with the complex mixed subtypes.

Different from the patients/advocates, the top two most important research questions identified by clinician and laboratory researchers were 1) determining mechanisms of endocrine resistance, and 2) identifying novel therapeutic targets, repurposing existing drugs and progressing them to clinical trials. These research questions align with the identified priority areas of ELBCC (https://lobsterpot.eu/organisation/working-groups and https://lobsterpot.eu/organisation/pr2), and likely reflect the desire to personalize therapy for patients with ILC through improved understanding of unique biology of ER and other signaling pathways. While preclinical and clinical studies have suggested that ILC may be relatively resistant to antiestrogen therapy^18–20^ and sensitive to inhibitors of growth factor receptor/PI3K/ROS1 signals^21–25^, clinical trial evidence supporting these concepts remain lacking. The first clinical trial that focused specifically on ILC (GELATO; NCT03147040) which evaluated chemotherapy followed by immunotherapy in patients with metastatic ILC showed a relatively low (27%) clinical benefit rate^26^. It was discontinued in part because the responding tumors were of the rare ER-negative subtype of ILC and because chemotherapy plus immunotherapy is now standard of care for patients with PD-L1+ metastatic TNBC, regardless of histological subtype. Of note, interesting correlative science from this trial as well as another recent study^27^ supports the need for further analysis of the immune infiltrate in ILC.

There are limitations to the study, which include uneven representation of the three stakeholder groups. In addition, the physicians are not truly a representative group as only 17% are in private practice suggesting limited participation from community physicians. In addition, physicians involved in the diagnostic practices, including pathologists and radiologists, are underrepresented in the survey respondents, which are skewed towards medical oncologists. As with many surveys, the responses are biased in terms of who takes the time to respond to the survey, ie the respondents are likely highly motivated to improve understanding of ILC. Finally, although we do have representation from 66 countries, almost two thirds of the responses (64%) originate from only three countries.

In summary, we have obtained data on world-wide understanding and interest in the study of ILC. Patients feel that communication of the unique features of ILC by the physician can be improved. While physicians and laboratory researchers feel the need to better understand endocrine resistance, to identify new treatment targets and/or repurpose existing drugs for ILC treatment, patients’ top priority research area is improved imaging. Overall, the survey indicates areas where interventions can be implemented to improve communication and outcomes for patients with ILC.

## Methods

### Survey development and measures

After discussing the concept and development of an early version of the survey, a draft was shared with representatives from the three groups, breast cancer clinicians/researchers, laboratory-based researchers, and the community including patients and patient/advocates (hitherto called “physicians,” “researchers,” and “patients/advocates”). Multiple rounds of edits were circulated among the team, and the survey was then further refined by the UPMC Hillman Cancer Center Population Survey Facility. After beta testing with a subset of physicians, researchers and patients/advocates, final edits were made, and the survey was developed and distributed using the Qualtrics online survey tool, through the Population Survey Facility. The final survey (Supplementary Text File 1) was fielded March to May 2022 to email lists compiled from PubMed, ORCID and professional networks, followed by distribution via social media.

For our study, we have complied with all relevant ethical regulations including the Declaration of Helsinki. The survey was anonymized, and responses were not linked to identifiers, however respondents had the opportunity to list names if they chose to be identified as survey participants in the resulting manuscript (Supplementary Data File 1). The study received Pitt institutional review board approval (STUDY21010058, initially effective on 03/22/2021, and final version was effective on 02/17/2022).

### Analysis of survey results

All responses were collected by the University of Pittsburgh Population Survey Facility. To analyze responses for topic of most critical and impactful research questions, each variable was dichotomized (most impactful or not), a top score was generated which was then analyzed using chi-square analysis across the subgroups.

Free text field questions 34 (“Which ILC cell models are you including in your studies”) and 58 (“Please indicate below other research questions that have high priority that we have not listed”) were manually summarized (SO, AVL) with common themes assigned for each answer with high consistency (overlap rate for Q58 = 98.36%). Natural language processing (Chat GPT, and sentence transformer – see below for details) was used to analyze responses to free text field questions 33 (“Why are you not including ILC cell models in your studies”) and 50 (“What do you now know about ILC that you wish you could have heard from and discussed with your…”), which were all initially filtered to exclude missing or semantically non-meaningful items. For question 33, a prompt was made for ChatGPT4, “Think as a physician scientist, patient, and basic researcher in breast cancer. Summarize the common but non-overlapping reasons shared among answers, ‘Why are you not including ILC cell models in your studies?’, For each reason, list only the respondent number.”, followed by respondent number and free-text answers in two columns, individually. The returned summarization and labeling of each answer were then manually inspected and adjusted (merging of reason 3 and 6). For question 50.1-5 (“What do you now know about ILC that you wish you could have heard from and discussed with your…”), all answers were combined (*N* = 1260), with each answer transformed into a 384-dimentional vector (embedding) via sentence transformers (https://huggingface.co/sentence-transformers), and converted to 5-dimension using UMAP with local neighborhood at 15. HDBSCAN was then performed with minimum cluster size of 15, on Euclidean distance and cluster selection method as ‘eom’, generating 23 clusters. Then for each cluster, class-based TF-IDF (c-TF-IDF) vectors were calculated among all words, followed by topic reduction via merging c-TF-IDF vectors with highest cosine similarity with 10 iterations, generating 14 final topics. The resulted topics were merged into 11 via manual assessment of words of top frequency within each cluster, after which all answers of the same topic, except for ‘ambiguous’ group, were passed to gpt-4 api deterministic model (temperature zero) respectively, with a prefix “Think as a patient with breast cancer, and as a physician scientist studying breast cancer, summarize in professional scientific language one single common topic shared among answers, ‘What do you now know about ILC that you wish you could have heard from and discussed with your breast cancer doctor?’ The summarization should be a single, brief sentence”. Then metastasis and recurrence groups were manually merged based on expert suggestion. This eventually led to 10 topic clusters, including one non-specific group (ambiguous).

Crosstabulations were analyzed using Chi-Square test, and for these some groups were combined (such as pathologists, radiation oncologists, gynecologists, and others) due to the limited number in such subgroups analysis.

### Supplementary information

Suppl material

## Data Availability

The study does not contain any sequencing or structural data. Majority of responses to survey as well as original survey document have been uploaded into Supplementary Text and Data Files. Additional raw data are available upon reasonable request from the corresponding author.

## Code availability

Codes for text analysis are provided in https://github.com/leeoesterreich/ILC-Survey.
