# Supplementary material for: International survey on invasive lobular breast cancer identifies priority research questions
Source: NPJ Breast Cancer. 2024 Jul 20;10:61. doi: 10.1038/s41523-024-00661-3 (PMC11271268; doi:10.1038/s41523-024-00661-3)
Supplement: Supplementary file 1 — Suppl material [file 41523_2024_661_MOESM1_ESM.pdf]

Supplementary Figure 1: Topics patients wished to discuss in more detail with their physicians.

A) Number of answers among topics for each specialty of physicians. B) Landscape of answers among topics in UMAP from semantic embedding

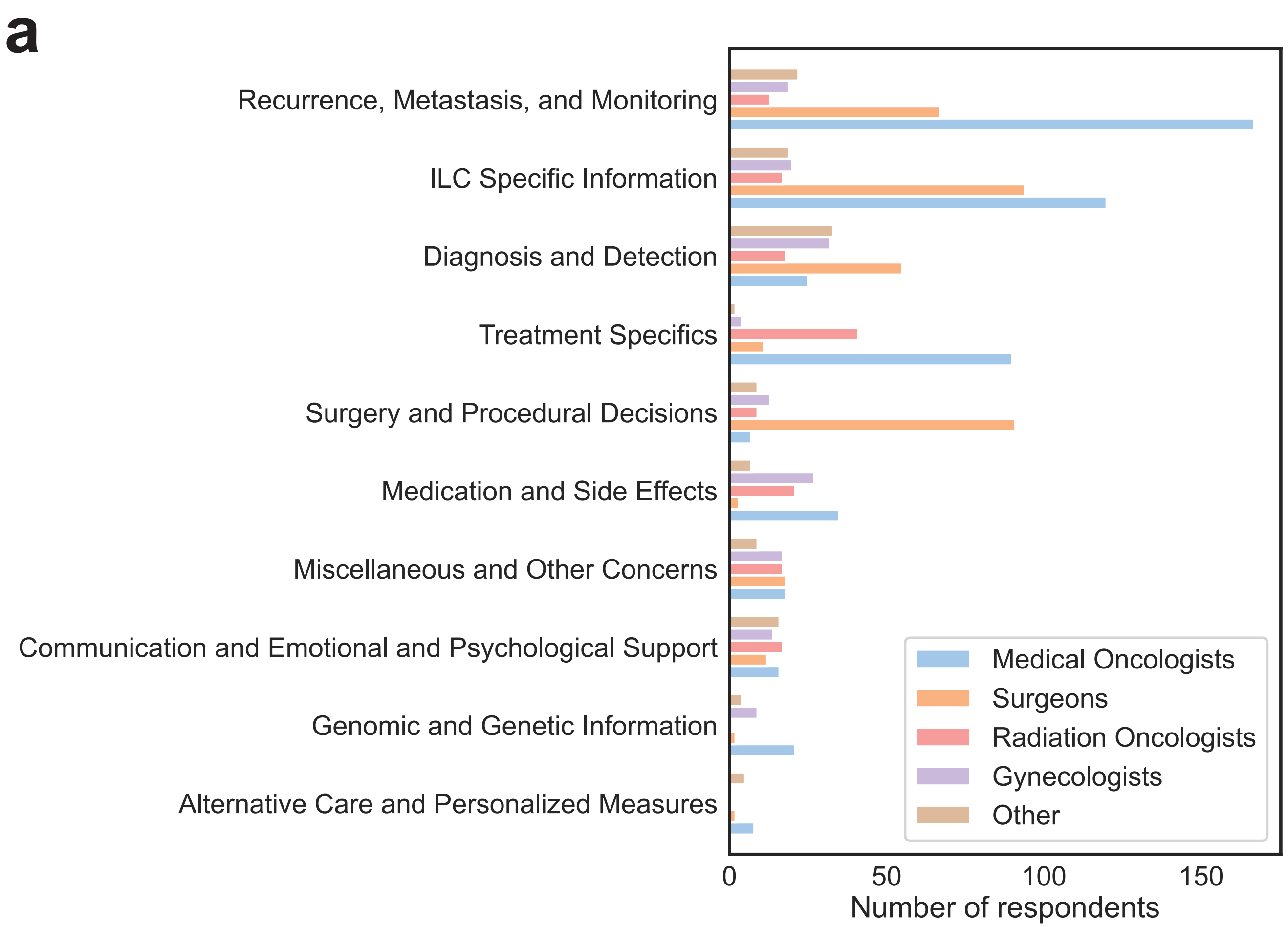

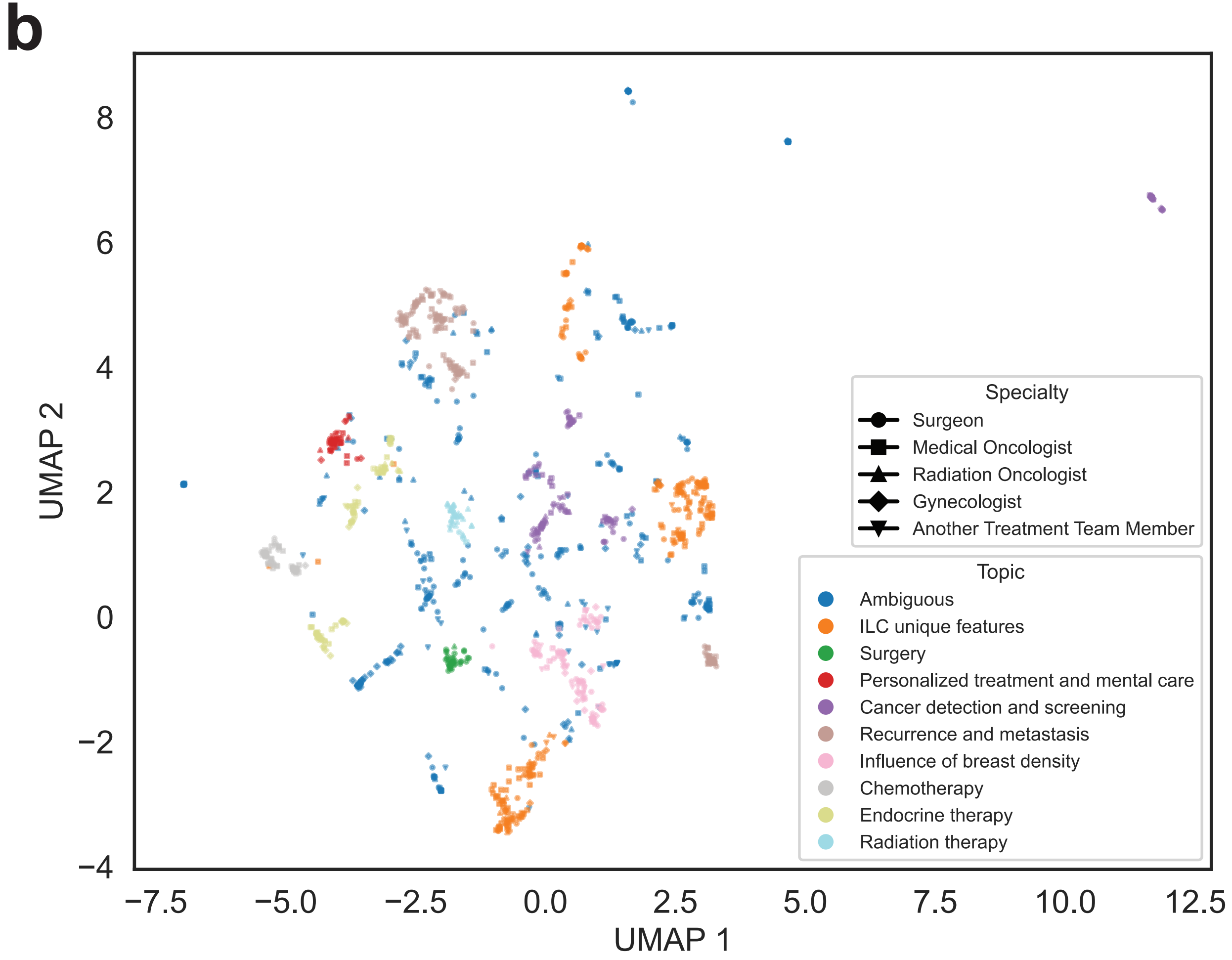

Supplementary Text 1

ILC SURVEY

Start of Block: Eligibility

Dear xxx

We are inviting you to participate in a voluntary survey focused on **Invasive Lobular Breast Cancer (ILC)** which was developed by an international group of researchers, clinicians, and breast cancer advocates. The goal of this survey is to understand current knowledge about ILC, and identify potential focus areas for future studies. We expect that the results of the survey will be summarized in a manuscript, and hope that they will guide future collaborative research. The survey was approved by the University of Pittsburgh IRB.

Please complete the survey by xx/xx/2022. If you have any questions about context, please contact Drs Steffi Oesterreich at oesterreichs@upmc.edu and Christine Desmedt at christine.desmedt@kuleuven.be. Questions from breast cancer patients and advocates can be sent to Leigh Pate at lpateseattle@yahoo.com, and if you have logistical problems with filling out the survey, please contact Dr Todd Bear at tobst2@pitt.edu.

You are receiving this survey since you have been identified as having published on ILC, having participated in prior ILC Symposia, being interested in breast cancer subtypes including ILC, and/or being a thought leader in breast cancer. We are not using any commercially obtained email lists. It is possible that you will receive more than one email with the invitation to participate in this survey, or you may see it posted on social media. You only need to complete the survey once. Please do not share or post the link to the survey you receive in this email.

You will fill out the survey anonymously, but there will be an option to leave your name, affiliation and contact email if you wish so that we can include your name when publicizing the results of the survey. Whether you choose to leave your name or not, your survey responses will be anonymous, ie your name cannot be linked to your survey responses.

Thank you from a worldwide group of scientists, clinicians and patient research advocates including members of the Lobular Breast Cancer Alliance (LBCA), European Lobular Breast Cancer Consortium (ELBCC), Lobular Breast Cancer UK (LBCUK), Lobular Ireland, and the Dynami Foundation who are interested in furthering ILC research.

Q1 Are you a physician or clinical researcher who treats individuals with breast cancer?

- No (1)
- Yes (2)

Q2 Are you a basic science/translational researcher actively involved in breast cancer research?

- No (1)
- Yes (2)

Q3 Do you have research focused on lobular breast cancer?

- No (1)
- Yes (2)

Q4 Are you **primarily** working in a...

- Academic institution (1)
- Private institution (2)
- Governmental institution (3)
- Other (specify) (4) ____

Q5 Have you had or are you currently living with breast cancer?

- ○ No (1)
- ○ Yes, I have had breast cancer (2)
- ○ Yes, I am currently in active treatment for breast cancer (3)
- ○ Yes, I am currently living with metastatic breast cancer (4)

Q6 Are you a member of a breast cancer advocacy group?

- ○ No (1)
- ○ Yes (2)

Q7 What is the **main reason** you became a breast cancer advocate?

- ○ I'm a caregiver for someone with breast cancer (1)
- ○ I'm a family member of someone with breast cancer (2)
- ○ Other (please specify) (3) ____

Q8 Which breast cancer advocacy group(s) are you a member of?

- ○ Advocacy#1 (4) ____
- ○ Advocacy#2 (5) ____
- ○ Advocacy#3 (6) ____

End of Block: Eligibility

Start of Block: Questions for physicians and clinical researchers

Q9 What is your **main** sub-specialty?

- ○ Surgical Oncology/or Breast Surgery (1)
- ○ General Surgery (2)
- ○ Medical Oncology (3)
- ○ Pathology (4)
- ○ Plastic Surgery (5)
- ○ Radiation Oncology (6)
- ○ Radiology (7)
- ○ Gynecologist (8)
- ○ Other (specify) (9) ____

Q10 How long have your practiced medicine:

- ○ Less than 1 year (1)
- ○ 1-10 years (2)
- ○ 11-20 years (3)
- ○ 21-30 years (4)
- ○ 31 years or more (5)

Q11 On average, how many breast cancer patients do you see in a month?

- ○ Less than 1 (1)
- ○ 1-10 (2)
- ○ 11-50 (3)
- ○ 51-100 (4)
- ○ 101 or more (5)

Q12 How confidently can you describe the histologic and molecular differences between ILC and IDC?

- ○ Not at all confident (1)
- ○ Slightly confident (2)
- ○ Moderately confident (3)
- ○ Very confident (4)
- ○ Extremely confident (5)

Q13 How important is it for you to know ductal vs lobular histology of breast cancer in your patients?

- ○ Not at all important (1)
- ○ Slightly important (2)
- ○ Moderately important (3)
- ○ Very important (4)
- ○ Extremely important (5)

Q14 Are there clinical trials or outcome data supporting unique treatment paradigms for ILC vs IDC?

- No (1)
- Yes (2)

Q15 How much does histology affect your treatment decision-making now?

- None at all (1)
- A little (2)
- A moderate amount (3)
- A lot (4)
- A great deal (5)

Q16 Would refined treatment guidelines specifically for lobular breast cancer be valuable for treating patients with ILC in the future?

- No (1)
- Maybe (2)
- Yes (3)

Q17 Why do you think refined treatment guidelines specifically for lobular breast cancer **would not be valuable** for treating patients with ILC in the future?

____

____

____

Q18 Why do you think refined treatment guidelines specifically for lobular breast cancer **would be valuable** for treating patients with ILC in the future?

Q19 How often do clinical trials and other clinical studies you are involved in collect information on histology?

- Never (1)
- Sometimes (2)
- About half the time (3)
- Most of the time (4)
- Always (5)
- *N/A, never involved in clinical trials or studies* (6)

Q20 How often do clinical trials you are involved in consider histology in the inclusion/exclusion

- ◯ Never (1)
- ◯ Sometimes (2)
- ◯ About half the time (3)
- ◯ Most of the time (4)
- ◯ Always (5)

Q21 Have you ever powered clinical trials you are involved in to do subset analysis of lobular cancer?

- ◯ No (1)
- ◯ Yes (2)

Q22 Would you consider powering clinical trials to do subset analysis of lobular in the future?

- ◯ Definitely not (1)
- ◯ Probably not (2)
- ◯ Probably yes (3)
- ◯ Definitely yes (4)

Q23 Please provide your reasons **for not considering** powering clinical trials to do subset analysis of lobular in the future?

____

Q24 Please provide your reasons **for considering** powering clinical trials to do subset analysis of lobular in the future?

____

Q25 Would you consider participating in a group consortium to conduct clinical trials on lobular breast cancer?

- ○ Definitely not (1)
- ○ Probably not (2)
- ○ Probably yes (3)
- ○ Definitely yes (4)

End of Block: Questions for physicians and clinical researchers

Start of Block: Questions for basic science/translational researcher

Q26 How long have you been working as a basic scientist/translational researcher?

- ○ Less than 1 year (1)
- ○ 1-10 years (2)
- ○ 11-20 years (3)
- ○ 21-30 years (4)
- ○ 31 years or more (5)

Q27 How much of your research is focused on ILC?

- None at all (1)
- A little (2)
- A moderate amount (3)
- A lot (4)
- A great deal (5)

Q28 Have you been funded to work specifically on ILC?

- No (1)
- Yes (2)

Q29 From what sources/organizations have you received funding to research ILC (Please specify below)?

____

____

____

____

____

Q30 How confidently can you describe the histologic and molecular differences between ILC and IDC.

- ◯ Not at all confident (1)
- ◯ Slightly confident (2)
- ◯ Moderately confident (3)
- ◯ Very confident (4)
- ◯ Extremely confident (5)

Q31 Are there clinical trials or outcome data supporting unique treatment paradigms for ILC vs IDC?

- ◯ No (1)
- ◯ Yes (2)

Q32 Are you including ILC cell models in your studies?

- ◯ No (1)
- ◯ Yes (2)

Q33 Why are you not including ILC cell models in your studies?

____

____

____

____

____

Q34 Which ILC cell models are you including in your studies?

____

____

____

____

____

Q35 Are there adequate in vitro and in vivo models of ILC for research?

- ○ No (8)
- ○ Yes (9)
- ○ No Opinion/Don't Know (10)

Q36 Is ILC adequately represented in large publicly available genomic data-sets?

- ○ No (8)
- ○ Yes (9)
- ○ No Opinion/Don't Know (10)

Q37 Are you able to obtain lobular breast cancer tissue and/or blood samples from patients with ILC for research?

- ◯ No (8)
- ◯ Yes (9)
- ◯ No Opinion/Don't Know (10)

Q38 What barriers or issues prevent you from obtaining lobular breast cancer tissue and/or blood samples from patients with ILC for research?

____

____

____

____

____

End of Block: Questions for basic science/translational researcher

---

Start of Block: Questions for Respondents WHO HAVE HAD or are living with BREAST CANCER

Q39
You indicated that you had or currently have breast cancer. The following questions about your cancer and treatment.

Which histologic type is/was your cancer (check all that apply)?

- ☐ Invasive Lobular cancer (ILC) (1)
- ☐ Invasive ductal cancer (IDC)/Invasive carcinoma of no special type (NST) (8)
- ☐ Lobular carcinoma in situ (LCIS) (15)
- ☐ Ductal carcinoma in situ (DCIS) (10)
- ☐ Hyperplasia or ALH (11)
- ☐ Mixed lobular/ductal (12)
- ☐ Other specific histology (13)
- ☐ Unknown (14)

Q40 With which stage of cancer were you initially diagnosed?

- ○ Stage 1 (1)
- ○ Stage 2 (2)
- ○ Stage 3 (3)
- ○ Stage 4 (4)
- ○ Unsure/Don't remember (5)

Q41 When were you first diagnosed with breast cancer?

- ○ Within the past 2 years (1)
- ○ Between 2 and 5 years ago (2)
- ○ More than 5 years ago (3)

---

*

Q42 How old were you when you were first diagnosed with breast cancer?

____

Q43 Has your cancer recurred?

- ○ Yes, locally or regionally (in the breast or lymphnodes) (1)
- ○ Yes distant, (in locations other than breast or lymphnodes) (2)
- ○ No (3)

Q44 Did your physician/health provider explain the unique features of ILC including how it presents differently from ductal?

- ○ No (1)
- ○ Yes (2)
- ○ Don't know/can't remember (3)

Q45 Did your care team discuss personalized therapy with you based upon your histologic diagnosis (i.e. IDC vs ILC)?

- No (8)
- Yes (9)
- I was not offered personalized therapy because my physician explained that the treatment is no different for ductal than for lobular (11)
- Don't know/can't remember (10)

Q46 Did you discuss ILC-personalized therapy with your...

	Definitely not (1)	Probably not (2)	Probably yes (3)	Definitely yes (4)
Surgeon (1)	○	○	○	○
Medical oncologist (2)	○	○	○	○
Radiation oncologist (3)	○	○	○	○
Gynecologist (4)	○	○	○	○
Another treatment team member (5)	○	○	○	○

Q47 Did your physician/ health provider explain how to monitor yourself for local or distant recurrence in the future?

- No (8)
- Yes (9)
- Don't know/Can't remember (10)

Q48 Did your physician/ health provider explain that lobular cancer cells can metastasize to unique places?

- No (8)
- Yes (9)
- Don't know/Can't remember (10)

Q49 Did your physician/ health provider explain what symptoms, including unusual symptoms, of recurrence you should report in the future?

- No (8)
- Yes (9)
- Don't know/Can't remember (10)

Q50 What do you now know about ILC that you wish you could have heard from and discussed with your...

◯ Surgeon (1) ____

◯ Medical oncologist (2) ____

◯ Radiation oncologist (3) ____

◯ Gynecologist (4) ____

◯ Another treatment team member (5) ____

End of Block: Questions for Respondents WHO HAVE HAD or are living with BREAST CANCER

Start of Block: ILC research topics

Q55
Please rate how critical and impactful the following research topics are with regards to **Epidemiology and Risk Reduction:**

	Less critical and impactful (1)	Moderately critical and impactful (2)	Most critical and impactful (3)
Understanding of the unique etiology of ILC, such as association with pregnancies, breastfeeding, alcohol intake, and hormone replacement therapies (1)	◯	◯	◯
Identifying strategies to improve ILC screening/early detection (2)	◯	◯	◯
Impact of obesity and lifestyle factors on risk of developing ILC and risk of relapse (3)	◯	◯	◯

Q51
Please rate how critical and impactful the following research topics are with regards to **Diagnosis (Imaging and Pathologic Analysis):**

	Less critical and impactful (1)	Moderately critical and impactful (2)	Most critical and impactful (3)
Examining use of E-cadherin/p120 expression, and/or other histological markers for routine diagnosis of lobular neoplasia including its variants (1)	○	○	○
Improving diagnosis and understanding of mixed IDC/ILC (2)	○	○	○
Understanding the use of artificial intelligence to improve diagnosis of ILC (3)	○	○	○
Understanding value of genomic predictors for ILC prognosis and prediction of therapeutic response (4)	○	○	○
Identifying strategies to improve ILC screening/early detection (5)	○	○	○

Q52

Please rate how critical and impactful the following research topics are with regards to **therapy, treatment resistance and disease progression:**

	Less critical and impactful (1)	Moderately critical and impactful (2)	Most critical and impactful (3)
Identifying mechanisms of metastases, and in particular, metastases to unique sites such as ovary and the gastro-intestinal tract (1)	○	○	○
Determining mechanisms of endocrine resistance in ILC, and potential differences to estrogen receptor action in IDC (2)	○	○	○
Identification of novel therapeutic targets and/or repurposing existing drugs for ILC and progressing them to clinical trials, including neoadjuvant trials (3)	○	○	○
Determining utility of immunotherapy in ILC (4)	○	○	○
Understanding value of liquid biopsies for diagnosis and monitoring treatment response in patients with ILC (5)	○	○	○
Characterizing chemotherapy in ILC, and understanding differences to IDC (6)	○	○	○
Determining mechanisms of dormancy and risk for late relapse (7)	○	○	○

Developing and testing lifestyle interventions (such as diet, physical activity) (8)	○	○	○

Q54
Please rate how critical and impactful the following research topics are with regards to **local therapy of the primary tumor:**

	Less critical and impactful (1)	Moderately critical and impactful (2)	Most critical and impactful (3)
Determining how to reduce the high positive margin rates in ILC (1)	○	○	○
Characterizing further whether breast conservation/radiation is as safe as mastectomy in ILC (2)	○	○	○
Determining how can we reduce the morbidity of axillary surgery, and whether radiotherapy can replace axillary surgery in ILC. (3)	○	○	○
Characterizing potential difference in post-mastectomy radiation between ER+ IDC and ER+ ILC (4)	○	○	○

Q53
Please rate how critical and impactful the following research topics are with regards to **Imaging:**

	Less critical and impactful (1)	Moderately critical and impactful (2)	Most critical and impactful (3)
Optimizing current breast cancer screening modalities (1)	○	○	○
Identifying new and specific imaging tools for ILC (2)	○	○	○
Studying the importance of breast density (3)	○	○	○
Determining the utility of MRI (4)	○	○	○

Q56
Please rate how critical and impactful the following research topics are with regards to **Lobular**

tumorigeneses (the formation of tumors), and other basic/translational research question:

	Less critical and impactful (1)	Moderately critical and impactful (2)	Most critical and impactful (3)
Determining cell of origin for ILC (1)	○	○	○
Focusing on development of a centralized ILC data and tissue registry (retrospective/prospective) (2)	○	○	○
Developing and characterizing ILC models (3)	○	○	○
Characterizing differences in the tumor microenvironment between ILC and IDC (4)	○	○	○
Understanding of LCIS as a precursor ILC (5)	○	○	○
Characterization of subtypes of ILC (pleomorphic, mixed etc) (6)	○	○	○
Understanding of the unique etiology of ILC, such as association with pregnancies, breastfeeding, alcohol intake, and hormone replacement therapies (7)	○	○	○

Q58 Please indicate below other research questions that have high priority that we have not listed.

____

End of Block: ILC research topics

Start of Block: Demographics

Q58 What is your Current age?

____

Q59 What was your sex at birth? Was it male or female?

- Male (1)
- Female (2)

Q60 Do you consider yourself to be transgender?

- Yes, Transgender, male-to-female (1)
- Yes, Transgender, female-to-male (2)
- Yes, Transgender, gender nonconforming (3)
- No (4)

Q61 In which country do you currently reside?

▼ Afghanistan (1) ... Zimbabwe (195)

Q62 In which state do you currently reside?

▼ Alabama (1) ... I do not reside in the United States (53)

Q63 What is your ethnicity?

- Hispanic (1)
- Non-Hispanic (2)

Q64 The last set of questions asks about if and where you see information about ILC.

Have you ever looked for information about ILC from any source?

- Yes (1)
- No (2)
- Don't know/Not sure (3)

Q65 The **most recent time** you looked for ILC information, where did you go first?

- Printed materials (1)
- Internet (i.e., published articles, websites, social media) (2)
- Interpersonal source (friend or family) (3)
- Health care provider (4)
- Information specialist (e.g., librarian) (5)
- Other (specify) (6) ____

Q66 Where do you go **most often** to look for ILC information?

- ○ Printed materials (1)
- ○ Internet: websites (2)
- ○ Internet: Social (Facebook, Twitter, etc.) (7)
- ○ Internet: Published articles (8)
- ○ Interpersonal source (friend or family) (3)
- ○ Health care provider (4)
- ○ Information specialist (e.g., librarian) (5)
- ○ Other (specify) (6) ____

Q67 Thank you for your time in completing the survey. We would like to recognize you for your time and effort by including your name in the acknowledgments section of any manuscript describing the results of the survey. If you agree with having your name listed, please insert your name, affiliation and email below.

- ○ Name (First, Last) (1) ____
- ○ Affiliation (2) ____
- ○ email address (3) ____

End of Block: Demographics

Supplementary Text 2

Why do you think refined treatment guidelines specifically for lobular breast cancer would be valuable for treating patients with ILC in the future?

Nevasse of different paterna of response to CT, diverse clinical outcome, Pattern of metastasis
Emlőmegtartó műtéttel több esetben megoldható lenne a daganat eltávolítása, mint ablatioval
Because ILC behaves differently from IDC
Different behaviour, different outcome require special guideline
Outcome for the patient. More objective for the physician.
A special type of breast cancer with differences in tumor characteristics, survival prognosis, and recurrence pattern from invasive ductal carcinoma
I'm not involved with making treatment decisions
Role of MRI in imaging; genetic testing and differentiation of prognostic subtypes of lobular carcinoma
Some of the biological differences affect the decisions around treatment. Eg - lymph node involvement often not detected preoperatively - is radiotherapy sufficient even if multiple involved nodes?
Target therapy/endocrine therapy
Not sure
Because recurrence style is differnt.
because it's harder to image effectively, has different relapse patterns that are good to highlight. Not a strong feeling.
Lobular carcinoma and ductal carcinoma are considered to have different biological characteristics.
Personalised therapy of lobular breast cancer is indeed a relevant issue from the clinical epidemiology viewpoint
Define patterns of recurrence. Personalized medicine for each pte.
Guidelines are most important to be follow by any medical oncologist. ILC need more guidelines
Improve treatment outcomes
Lobular cancers tend to be more responsive to endocrine therapies and tend to have less responsiveness to chemotherapy. Oncotype and other prognostic tests do not really adequately represent ILC. Management of pleomorphic ILC is I think the most challenging with no clear guidelines of global experience summary to rely on
This sub-type has a distinct histology, presentation, diagnostic issues, and natural history
ILC presents a different outcome in comparison with other BC histotypes. Lymphonde metastasis and recurrence are higher in ILC, and chemotherapy and well endocrine therapy are insufficient to control the progression of ILC. We need of targeted therapies for ILC, as trastuzumab for HER2 positive BC.

Several reasons - The lobular group is fairly large, and clearly a distinct entity. We wouldn't treat breast cancer like colon cancer (without evidence to do so), so why would we do it with lobular vs ductal? Additionally, it would draw attention to these distinctions - ignoring them is fairly ingrained. This is especially important in research, which often does not separate out the lobular group, considerably muddying the interpretability of results. Third, it would help patients to recognize that knowing their histology is important to appropriate treatment, expected course of the disease, etc. Fourth, it would foster development of appropriate research lines identifying most beneficial paths in treatment.

More tailored treatment and hopefully better outcomes

more mast4ctomy as a first operation less NACT CDK inhibitors

because they take into account the immunohistochemical characteristics

Precise medicine is the future.

Maybe more like precision medicine

Difference in prognosis

Yea

Lobular carcinoma is more likely to metastasize distantly than ducal carcinoma, so the rate of chemotherapy tends to be higher, but we believe that it is necessary to extract finer stratification factors for the risk of recurrence.

ILC patients exhibit different response to different therapies as compared to IDC.

There are specific guidelines for the treatment of ILC

Looking forward to the conclusion

Lobular carcinoma is different from ducal carcinoma.

生物学活性不太一样的肿瘤治疗方式应该不同

Due to the differential response to endocrine and potentially other therapies.

Cause therapy for other invasive cancers doesn’t work for ILC

Unclear if chemo plays much if any role in lobular. Unclear if axillary management applies. Unclear if size for PMRT matters (volume of disease vs length).

Pronostic life patient

.

Different molecular characteristics that may lead to different biology and treatment requirements

It may be. I don't really know.

Yes

New treatment lines are revolutionary

Depending on the subtype of ILC, different locoregional strategies are now being investigated (for example: preoperative radiotherapy). Not only in terms of biological and clinical behaviour, but also in terms of radiological appearance, ILC comprises a truly separate entity which demands refined treatment guidelines.

because ILC usually has a different clinical behavior, especially in advanced disease, and treatment needs to be individualized

My clinical experience indicates that it is different from IDC

Will encourage individualize treatment plan

if they have better outcomes than if they got the standard IDC treatment

To reduce variation and improve treatment.

it will be valuable if specific predictive factors for ILC will be discovered

Nehezebb felismerni, vékonytű, vagy core biopsiával. Gyakran több gócú, Sokszor már hónalji áttéttel jelentkezik.

Yes

Their histology and prognosis is different, yet are lumped together.

It's important to compile all data from these patients, especially those included in clinical trials, evaluate treatment and outcomes to build an international guideline. Also, would be very useful to build a virtual training and standard procedures to pathologists working in developing or low income countries which need assistance to ILC pathology diagnosis.

Because they have distinct molecular features

Some patients may be candidates for treatment de-escalation without the need for chemotherapy. Even personalization of surgical treatment.

Prognosis of ILC is far better than IDC , in Resource poor country where Chemosensitivity and molecular diagnosis are not done for precision therapy , many patient are exposed to the side effect of general chemotherapy for Breast cancer , treatment tailored guideline will be of benefit to patient with invasive lobular which could by diagnosed by histological pattern and IHC absence of E-cadherin

due to its difference in outcome with IDC

Some important questions arise when treating ILC. In the early setting, some examples are remarkable: the choice of the most adequate treatment regimen, the value of genomic testing, and so the treatment options may in fact vary when ILC is present, and several doubts on how to decide remain. Refined treatment guidelines would certainly reduce heterogeneity and uncertainty regarding these and other decisions for these patients.

The surgical technique should differ from ductal carcinoma of the breast depending on the mechanism by which lobular carcinoma spreads within the breast. Evaluation of axillary lymph node metastasis is also difficult for lobular carcinoma, and we believe that axillary lymph node management also requires its own guidelines. Regarding drug therapy, lobular carcinoma can be divided into classical and pleomorphic types, and HER2 mutation is often observed in the pleomorphic type. Therefore, it is important to use a different strategy for drug selection than for ductal carcinoma.

different biology mutations), clinical characteristics different metastatic patterns Need more accurate imaging

I believe in increasing personalization

From a med onc point of view, specific guidelines may become important if clear prognostic or predictive value of lobular vs non-lobular will be established. In addition, better imaging of ILC, both early and advanced will be valuable

because the evolution in the timeline is different.

as the differences in treatment may emerge more over time and currently this is suggestion that differences may improve outcomes

I belive that chemo is less efficient in lobular histology.

Beause ILC give metastasis mostly OUT of Lymph Node

in terms of breast surgery choice sometimes there might be concern specifically for breast conserving surgery

Because of its tendency to be multifocal and bilateral and difference in response to chemotherapy compared to ductal

I believe most oncologists do not take this histology into account

Because IDC and ILC are different diseases

Maybe less patients would receive chemo

ILC has worse prognosis than IDC

We need to tailor treatment specifically to these unique subtypes to acheive the best outcomes

They May be hepfull

We can avoid chemotherapy

I have many patients with ILC

We Need more precision medicine, and We have tô consider the diferences in responses between ductal and lobular cancers

It is a different disease with different responses to therapies (less responsive to chemo).

De escalation therapy

Lobular is a diferent disease and it needs tô be recognized in guidelines

It's a different histology with different molecular biology. It's very quite necessary to have specific data about ILC to help us to guidance the treatment.

To de-escalate chemotherapy

we need more clinical trials in order to define a guidelines

This is a chemo resistant histology but clinical trials are needed

Because outcomes are totally different!

prognosis evaluation and more personalized treatment

Tailored treatment considering the advances in the field.
Different behavior, more hormonal sensitive, less screenalbe with US and MMG
Specific treatments and prevention and early detection. New assessments tools for detecting Lobular
Duration of adyuvant treatment
DEEPER KNOWLEDGE OF THE ILC DISEASE IMPROVES THE PROGNOSIS AND QUALITY OF LIFE OF PATIENTS BECAUSE WE ARE ALWAYS MORE SELECTIVE AND LESS TOXIC, OFFERING DIRECTED AND SPECIFIC TREATMENTS.
MR staging
choice of endocrine therapy and need for chemo
because ILC often gives distant metastasis many years after diagnosis
beocause of differences in biological behavior of the cancer, less chemosensitivity and because of different local treatment(surgery and radiotherapy)
Lobular cancer has different patterns of metastasis and bilaterality unlike IDC.AI has more response than Tamoxifene
help tailor diagnosis and treatment and follow up
There must be some difference we are yet to know.
ILC have diferent behavior
Because ILC has a different clinical behaviour.
It will be important to join "omic", epidemiology information, and to know long term natural history. (bilateral breast cancer, peritoneal and ovarian pattern, link with digestive cancer, etc)
Because you can determine better what kind of therapy each patient should receive
Since lobular breast cancer differs in sensitivity to chemo- and endocrine therapy.
because of the differences for clinical behavior
It is important for the peculiarity of this disease
If we have a refined guideline for patients with ILC, that means that we would have strong supporting data.
It would help us in property advising our patients in this subgroup better and also aid in prognosis and perhaps the right targeted treatment to use.
A műtét típusának, terjedelmének meghatározásában.
Lobular breast cancer is a special entity
to make the best treatment desision
most of this cases need mastectmoy .
More information helps to tailor better treatment for patients- not only lobular/ductal or rare subtypes, but also mol. biology - NGS results

These guidelines would be based on evidence and expert opinion and.hopefully improve patients outcome

Some variant of ILC has bad prognosis , so better treatment dedicated specifically for ILC would be valuable

sparing unnecessary treatments and side effects

Limited Data.

ILC is a different disease than NST and should be managed differently from diagnosis (different imaging techniques) to type of local and type of systemic therapy in both early and metastatic settings

Fontos

because of they are harder to identify om mammograms and MRI, because they respond differently to neoadjuvant therapy and still they usually do not have a worse prognosis than invasive ductal carcinomas

Because there is growing evidence that these are two distinct entities

because this is a different setting

Since I am a radiologist, my answers are more aimed at the diagnostic part. Obviously we already have a sort of standard indication for MRI in these patients. Also my reporting of subtle findings will be adapted to the histology (i.e it is essential information for an application). NAC evaluation in these patients is very difficult and also here protocols could be adapted to the underlying histology. Likewise, since metastatic patterns are different (and quite often mets are not PET positive), for search of distant disease, protocols might be adapted

it is a distinct biological entitiy with different outcome and therapy response

Because currently there is no difference in recommendations according yo histology and more and more data is coming for example for the response of ILC to chemotehrapy.

Because it is a totally different compared to IDC

-

Because ILC has unique molecular features.

As a radiologist, I consider it important to discover ILC in screening tests, not just on the basis of clinical symptoms.

A need to clarify the differences between ILC and IDC and their practical consequences

This is a different disease with different progression dynamics and different metastatic features from NST carcinomas.

Till now the immune response as well as the protein translation efficiency and metabolism of either Timor is different and needs further research that would ultimately lead to more refund treatment.

There are many unique features about ILC, and its variants. Like response to chemotherapy, sites of recurrence, clinical behavior, etc

Moving the needle forward for precision medicine Meeting unmet needs for ILC patients and addressing over- and under-treatment for these patients (women)

yes

If it improves detection of additional cancer and/or improves outcomes

Better outcomes

ILC tend to be grade 2 T1-T2 but when larger or occasionally grade 3 or for example, histiocyrpid variants, it may seem logical for neoadjuvant but then response may be suboptimal so there is need for more data in ILC

yes

Because the risks associated ved ILC are bigger than for IDC, and the effect of chemo may be less

Better to address the roles of partial breast irradiation and the unusual patterns of lobular mets partic wrt surveillance protocols

different management mandatory

More research into Genomics profile of ILC and data on response to neoadjuvant chemotherapy, in particular.

Because ILC pt sometimes follow different course from IDC pt.

ILC appears to be biologically different from IDC.

Lobular cancer has some specific behaviors that differ from IDC that warrant specific treatment/management considerations

Behaviour Bilaterally Multicentricity Masking radiologically effect

It would be valuable because it is a disease with different clinical presentation, disease trajectory and sensitivity to treatment.

ILC is under represented in most trials. Should we be approaching them differently as far as imaging, chemo, rads, etc

not sure. I do not treat the patients.

Because of the different and discontinuous growth, the likely earlier and more aggressive metastasis and different treatment options.

Approach to staging

Current treatment guidelines are based on invasive ductal carcinoma of the breast, and may not apply to invasive lobular carcinoma.

While ILC is not nearly as common as IDC, the presentation of ILC on imaging and the nodal burden that we often find that can be imaging occult makes treatment sometimes more difficult. And the systemic treatment recommendations are often made as if patients with ILC will respond the same as patients with IDC, which is often not the case.

As ILC has different patterns of metastases and appearance on FDG and FES PET, Histology specific guidelines involving Selection of Radiology studies would be beneficial for patients.

The clinical/pathologic features of ILC can be quite different than IDC in terms of the difficulty in determining tumor size with conventional imaging/image occult disease, not uncommon extensive pathological nodal involvement without any clinical evidence of nodal involvement, and the rather indolent growth pattern and HR positivity, yet sometimes aggressive metastatic potential.

Less responsive to chemotherapy and possibly more endocrine sensitive

Sometimes I wonder I should do adjuvant chemotherapy for patients with high grade ILC.

Lobular cancer tendencies and behavior is different from ductal carcinoma

If it improves outcomes

On determining when and where reconstruction fits into the spectrum. For example, delayed oncoplasty versus immediate,

Anecdotal experience that imaging is less reliable, little/no response to chemo. Would be great to quantify this and offer more data to help with decision making

Difference of metastatic pattern between lobular cancer and other type

Because it is a histological type that accounts for a large proportion of special types of breast cancer and differs from IDC in terms of the mode of tumor progression, etc., individualization of treatment may be useful.

It differs from other kinds of breast cancers, in terms of growth pattern and often also in terms of aggressiveness, further it can be difficult to diagnose at mammography and perhaps also at ultrasone and MRI

yes

Treatment guidelines are very important so we can examine pathways

chemotherapy resistance new drug on trials (antiROS1)

Of course.

The ILC biology is different from IDC, thus the treatment should not be the same. Moreover, the natural history with potential metastatic sites justifies a different workout to assess tumour dissemination.

It will give better guidelines about what to do. Mastectony vs BCT and what kind of radiology that is nessesury

Not only IHC subtype, ILC would be an important biomarer to decide the treatment.

because in some indications it differs from IDC

I believe the biology og ILC is distinct from other histologic subtypes, and therefore specific treatment guidelines may improve outcome

Different margin requirements at lumpectomy

ILC behaves differently than IDC, it also has differences in genomic aberrations (for example higher rate of HER2 mutations), it tends to be less responsive to cytotoxic chemotherapy and it is more difficult to detect radiographically. In summary, it is DIFFERENT than IDC.

Patients may not respond as well to chemotherapy compared to IDC ER+ patients. Patients with ER+ ILC are lumped in with ER+ IDC guidelines and may have not been as well represented on the trials that led to these guidelines

N/A

It is difficult to define the number of LN metastasis

ILC cancer is biologically distinct from IDC and its pathogenesis is different. I think in the future we should and will focus on more targeted diagnostic and treatment options in ILC.

selective new treatments-kinase inhibitors hormone responses and combinations
?
De-escalation of chemotherapy in some patients
different biology, need for more personalized treatment
Avoidance of chemotherapy by estimating the specific contribution of ILC histotype independent of signatures such as OncotypeDX
Better outcomes are likely because the molecular biology and clinical behaviour is different from invasive breast carcinoma of no special type
Because of treatment resistance. Especially locally advanced disease
Hopefully we soon get solid evidence for specific treatment of ILC vs other BC histological subtypes.
To know the prognosis
Because in our own practice the relative number of pts with ILC that have ab initio or develop stage IV disease is (relatively) increasing
ILC is an other type of breast cancer with different molecular characteristics
Because patients with ILC tends to "drown" in the ocean of ductal breast cancer. Treatment decision making is mainly based on knowlege learned from BC studies including a vast majority of ductal cancers
According to the WHO histopathological classification, invasive breast cancer is divided into two main groups: special and non-special types. Invasive lobular carcinoma is one subtype of this special type and accounts for about 5-10% of all breast cancers. While most invasive breast cancers originate from the ductal epithelium, invasive lobular carcinoma is thought to originate from the lobules near the terminal mammary ducts, thus differentiating its biology from that of other invasive breast cancers. Invasive lobular carcinoma lacks molecules involved in cell-cell adhesion and maintains a sparse density of cancer cells, leading to progression and metastasis. As a result, they may extend more widely than preoperative imaging assessments suggest, and have the strange recurrence which is not usually observed. Translated with www.DeepL.com/Translator (free version)
Targeted treatments
Less chemo sensitive, MMG and US occult disease so MRI guidelines would be useful.
ILC is usually shows high endocrine responsiveness, and low chemo response. It may be multicentric although it maybe treated with breast conserving surgery. In large ILC tumors, neoadjuvant endocrine therapy is a very useful tool
It will lead to better outcome of our patients
Recurrence, chemotherapy options, predictive markers
It’s helpful to have all the highest quality day gathered in one place functioning as evidence based guidelines for every subtype of disease
Different biology and prognosis

Lobular breast cancer has a different metastasis pattern and poor sensitivity to chemotherapy. It should not be treated the same as ductal just as triple nwgative should not be treated like hormone positive breast cancer. It is a unique entity.

It would be useful to have an "oncotypeDX" specifically for ILC. It would also be helpful to develop specific guidelines for ER+/PR+ and ER+/PR-neg ILC

1. Screening: If we can identify risk factors for lobular cancers, we may consider either adding MRI or additional imaging modalities for timely diagnosis. 2. Adjuvant treatment: Genomic studies are not very helpful in identifying who benefits from chemotherapy in ILC. Redefining this and having a different cut-off for ILC specifically would be helpful. 3. Adjuvant therapies: Role of novel targeted therapies and IO needs to be explored in these tumors 4. Metastatic disease; Involvement of sites such as bowel/ovaries makes it difficult to detect and monitor metastatic disease in mILC. Use of FES-PET scans and other novel modalities needs to investigated. 5. Agents that can affect/impact the disseminated tumor cells in bowel and peritoneum is needed. 6. The big challenge we face in ILC is dormancy and late recurrence. Understanding dormancy and how to get rid of these cells will be critical for improving overall survival in these patients.

Currently most of the studies that define treatment for invasive breast cancer have lumped lobular in with ductal. While there have been retrospective analyses separating out ILC from IDC, there are not high quality prospective trials of treatment for ILC specifically

Lobular cancer represents a peculiar entity of breast cancer, different from ductal histology

Different response to endocrine and chemotherapy, different relapse pattern

To help guide decision making based upon what is known about the unique biology of lobular cancers and how to best manage them.

because of their specificities (biological and clinical), in order to improve the long term prognosis of ILC patients

This would provide a basis for more mechanistic based therapy.

Yes

improve diagnosis

So that we can achieve more accurate imaging, better surgical outcomes, more precise risk stratification, prediction of treatment response, reduction in harm from ineffective systemic therapies, reduced recurrences.

Need ILC specific guidelines around chemotherapy in both the early and late setting

Sentinel lymph node biopsy after systemic therapy. Local staging: MRI yes/no.

The prognosis and disease course is different with IDC.

Because lobular more often gives recurrence and cancer in the contralateral breast

differential responses to chemotherapy potential for targeted therapy exploiting molecular features of ILC

Because ILC show a distinct morphology with different longterm outcome and different location of metastasis.

If treatment guidelines should be refi Ed for Libular than they should be published to ensure lobular patients are getting the best specific care possible.

to refer to for diagnostic approach and treatment

They all need mri, they need different chemotherapeutic agents and more understanding of the E cadherens

To stop over treatment

Metastatic lobular cancers are more likely to be in effusions and on serosal surfaces, GI tract, bladder, etc, where drug delivery may be less effective. New regimens that focus on these differences could be helpful (intraperitoneal delivery, etc.). If some drugs work better than others in the more diffuse lobular cancers, then lobular specific guidelines may be useful.

different disease and risk factors

Understanding the value of chemotherapy in high genomic risk ILC patients

Different metastatic behavior

It might improve outcomes

Because the disease is morphologically, clinically and molecularly distinct from carcinoma-NST. It is highly likely that more targeted systemic therapies, directed at distinct molecular features of ILC, will improve outcome of ILC patients. In addition, surgical decisions may change as soon as it considers ILC. Since ILC often disseminates to the GI tract, GI complaints should be approached more aggressively in ILC than in carcinoma-NST patients to rule out M1 disease.

To personalize care of breast cancer.

Different clinical, pathological and molecular features

Lobular breast cancer has proven to be a seperate entity. It requires specific diagnostic measures like use of an MRI. Differences have been seen in treatment response and therefor seperate guidelines would improve the care of patients with ILC

Major risk of under treatment

if there are distinct differences in treatment that knowledge would be valuable and should be disseminated

Because invasive lobular carcinoma infiltrates and metastasizes in specific ways that are unlike invasive ductal carcinoma

Our research shows that ILC can have unique aberrations in signaling pathways different from the invasive carcinoma of no special type.

ILC do not respond similarly to standard treatments for breast cancer, NOS and should not be lumped together as if they do

ILC has many characteristics that should change treatment from IDC. For example, sensitivity to drug, positivity of SLNB, positive rate of truncation at surgery etc.

personalised cancer treatment

it is often detected at a later stage as it is very difficult to detect on screening mammogram. It is usually palpable. It is bilateral.

local regional therapy may be different

It would lead to better treatment outcomes

Hopefully there will be different treatments in the future

ILC has its special biology. For locoregional treatment en systemic treatment it has its own 'rules'.

We need to define novel treatment strategies for this subset of patients.

Because of known differences in tumor biology, hereditary factors, detection, preoperative diagnostic, follow-up, amount of surgery, endocrine therapies

If therapeutic targets unique to ILC were identified it would be valuable

This is a unique histologic entity compared to IDC and deserves a more targeted, ILC-specific approach.

Depends

Because of the different histomology en prognoses, also to specify therapies for their most likely respons

Nodal metastases are quite different compared to IDC (single cells vs clusters). Patterns of spread different. Often times ILC not well delineated on standard breast imaging and more extensive disease found at time of surgery

Subtle differences in approach

It is a very chemo-resistant disease, so knowing the clear path will help.

Since it differs from other BC subtypes

The current WHO definition of ILC is ambiguous and needs to be updated in order to get a better understanding of the natural history and optimal treatment and surveillance of patients with ILC. I would include that also a better understanding of LCIS is needed.

My research studies ground my thinking that the fundamental differences between ILC and IDC likely provide different windows of therapeutic efficacy, particularly for treatments that may impact invasive potential (an area of focus for my lab).

could highlight some specificities (if any in routine care ?)

Because physicians often wonder whether patients with ILC should be treated differently and would appreciate more guidance.

Benefit of different treatment modalities probably varies according to subtype

Because there are many specifics in ILC that deserve to be specifically adressed

Because ILC is a distinct molecular, pathologic and clinical entity from IDC.

Because i think it is different the other type breast cancer

Emphasize to those not completely specialized in breast ca what is actually known about lobular ca and how it differs from ductal and levels of evidence.

type and duration of endocrine therapy

Molecular testing such as oncotype dx is still ordered routinely despite evidence to support that is not necessary in classical ILC Margin decisions based on LCIS with variant histology is not uniform Clear definitions of ILC lymph node mets due to the unique single cells or shotgun patterns that are now judgement calls for pathologists

Treatment paradigms for breast cancer are rapidly shifting and the current classification of HR+/HER2+/TNBC will soon be insufficient. Patients with ILC often relapse late despite being responsive to endocrine therapy. In order to deepen response and improve rates of cure, future treatment guidelines for ILC will need to steer clinicians to target the unique molecular drivers in ILC.

Differing response to therapy and differing underlying biology

- Improve outcomes - Refine work-up - Spare unecessary toxicity

more precision

Surgery, chemotherapy , neoadj endocrine therapy esp in premenopausal women

Mainly for work-up: MRI needed to determine disease extent in ILC, consideration of maybe mastectomy over wide local excision, follow-up with MRI

ILC is a unique cancer with different biology, presentation and recurrence profiles. It responds differently to traditional chemo and endocrine therapy.,

Different disease, different treatment options

ILC evolution is different from IDC and should treated differently

ILC patients have different growth pattern resulting in delayed diagnosis, different genetic characteristics (driver mutations and CDH1), different metastatic pattern, different survival rates (poorer 10 year DFS, OS) and despite of all these differences the treatment is very similar to IDC apart from neoadjuvant treatment (chemotherapy) which is not offered to ILC in Denmark. it would be great with joint randomized studies for ILC patients.

I believe that ILC represents an area of huge unmet needs. ILC has a different clinical behavior compared to IDC, but nevertheless patients with ILC and IDC are treated with the same drugs and are included in the same clinical trials. Moreover, several clinical trials do not even provide subgroup analysis according to histotype, thus leaving uncertainties on the specific benefit of treatments in ILC.

ILC has unique biology and would benefit from more targeted approaches. In the adjuvant setting, for instance, not all gene platforms perform equally in terms of prognostic information and need for adjuvant chemotherapy, and this is of paramount importance for treatment decisions.

yes, based also on different ILC types.

Local therapy is affected by lobular histology

subdivide ILC subtypes

I'm in favor of conservative treatment for ILC, but there must be more cautious regarding the risk of multifocal, multicentric or bilateral breast cancer.

Contrast of endocrine vs chemo-sensitivity; unique metastatic patterns and presentation

Please see previous comment

current guidelines do not recommend different treatment approaches by IDC and ILC histology, yet there is ample but inconclusive data which suggest that these distinct histologies may have different chemotherapy sensitivity and recurrence pattern.

Because current guidelines are limited in their description on histology, I want to know comprehensive treatment strategies for ILC, even if they are based on expert opinion.

improve outcomes

Different clinical presentation and different clinical evolution

- helpful for identification and screening (not a lump, often not found by mammo.) - mets. tends to occur later than with ductal - aromatase inhibitors are more effective than tamoxifen - mets. found in different areas than ductal ie., peritoneum, orbital, etc.

I think issues of initial detection and surveillance imaging are distinct for ILC vs. IDC.

Please see prior question. (molecular targets)

The decision about whether to give chemotherapy to ILC patients is frequently a tough call. Having guidelines that provide recommendations on this would be very helpful. Other issues that guidelines could help on include adjuvant abemaciclib use for ILC patients, and differentiating classical ILC vs high risk ILC subtypes, such as pleiomorphic ILC.

To improve personalized approach to therapy. Because ILC and NST have different biology.

Most ILC are hormone receptor positive and many present with large tumors. pCR rates are low in this setting and chemotherapy does produce some clinical response but not pCRs. It would be great to know which patients should be treated with chemotherapy up front and to have guidelines for that.

as i said, maybe

If there are treatment differences, this would be valuable.

Important differences include the best ways to monitor treatment for ILC and also understanding the unique molecular differences that can guide treatment in the metastatic setting. Therefore, if true actionable mutations/pathways are discovered and incorporated into an algorithm, its possible that ILC patients would do better overall.

Specific growth and metastatic spread

Because the patterns of progression is quite distinct between ILC and IDC.

Precision medicine

It appears to be a distinct histological type and although largely hormone receptor positive behaves differently.

Supplementary Text 3

Please provide your reasons for considering powering clinical trials to do subset analysis of lobular in the future?

Please provide your reasons for considering powering clinical trials to do subset analysis of lobular in the future?
As molecular biólogy is not the same, I wonder response is not either
Different response to special therapies
Investigation of the patient(MR? Contrast mammografi), neo adjuvant or not, what kind of operation type?
there is obviously some need to look at this data
Subset analysis will be the primary way that definitive answers regarding unique information will be collected
To see there molecular profile in subsaharan africa
Since my activity in the Lobsterpot COST action I will focus my interest over this topic
Important for prognosis
Breast cancer is one of my area of research and powering clinical trial of ILC in my country is definitely one of my dream
Increase specific information that will guide treatment
Lobulars often show different types of response than IDC to therapy, especially chemo
It is the only way to quantify the best treatment approaches if they differ between histologies
ILC is a clear different sub-type of BC. Molecular biology, prognosis and survival is different from other BCs.
Essentially the same as my prior comments. It is a distinct entity. If adequate research is not done to identify informed approaches to management, then we are treating "in the dark." That does not make sense (even if it is painful to recruit sufficient quantities of patients with this cancer, due to the relatively lower numbers involved). Further, it would disentangle the awkward basal vs luminal categorizations, and similar lumping of all HR+ brst cancers together as is common in much of the breast cancer research.
different presentation, different drugs
It's rare, but worth to be noticed and understood.
Difference in prognosis
It's important
Because I am interested.
ILC May have different response to Immunetherapy

Clinical, histological and evolutive characteristics of ILC are most of time different from other histological types

Pathological results guide clinical practice, so I fully believe that more systematic and targeted treatment guidelines can be published in the future to better improve patient prognosis and precise treatment.

Different biological behavior

从病理上是一个很好的亚组

Better chance of treatment

Need specific data - can’t assume IDC data applies to ILC

Treatment patient

.

different characteristics require specific analyses

To support research for new treatment protocols

For proper treatment plan management

Hope for better treatment lines

It is only by capturing the different characteristics of lobular carcinoma (in contrast to other histologies) that progression in future treatments will be possible

I think it can give us valuable information about future treatment guidelines

Lack of information of lobular carcinoma

To further advance knowledge about different tumorbehavior acoording to histologic type.

may be they could have completely different behaviour from IDC

A teljes emlőrák diagnosztika szempontjából fontos. A túlélés szempontjából fontos.

For better treatment

I believe the best way to do it is though big data/ real world data analysis; not per clinical trial.

If we intend to personalize the treatment of patients with breast cancer, we must consider the issues of different histological presentations.

Breast cancer generally is the most common malignancy among female in Nigeria and my institution been a regional centre for oncology in Nigeria have a cohort of Breast cancer patient that may benefit from such clinical trial

due to the lack of data so far

In order to improve evidence for these histologic subtype of BC, we need to have robust data to support our practice an powering trials for this subset is certainly a way to go

Better understading

Ductal and lobular carcinomas have different biology

ILC can have sometimes different specific metastic sites (pleural, peritoneal)

As mentionned before probably different disease

I believe in increasing personalization

if there were a hypothesis particularly focused on lobular histology subset

I belive IDC and ILC behave differently,

Métastasis Pattern of Invasive Lobular Carcinoma

since it is a different entity comparing to IDC it would be reasonable to check it accordingly

Difference in response compared to ductal

So we can draw conclusions

Because I do expect different outcomes

It would be possible to personalise treatment if we understand better the way different hystologies respond to treatment

it is different entity

Clinical trials are the bedrock of changing and improving clinical practice and treatments

It is a different concept and dinamics

To assess differences in response to systemic treatments

its important the diferences of the subset for treatment.

There is a different Breast câncer

Different disease with different drivers and responses to therapies.

It would help in clinical management

To understand the differences and how does it impact the prognosis and the survival of these patients

The biology os different

in order to have better knowledge on how to treat these patients

To better stratify the histology

To improvement management

Improve tailored treatment

They have different behaviors

I

Is more frecuently in the ordinaey clinical practivw, and the relapse is presenting ultil 10 years after
It could be difficult because ILC represents a small percentage of all types of breast cancer
because ILC behaves differently than IDC
because of differences in outcome and activity of drugs
Different pattern of metastasis and response
to help tailor results in this area and inform treatment
To find the difference in results to enable us for specic and directed study for ILC
X
ILC is out of the most recent tests and trials.
Because could be a window of oportunitty to understand lobular ca
More precise results for this subtype
the need for treatment recommendations specifically for lobular breast cancer
biological behavior
For the tumor characteristics and the endocrine sensitivity and also for the different spread in metastatic setting
ILC a clearly distinct tumor type compared to IDC. If this statement is true, we need clear clinical, and translational data on the effectiveness of the available treatments, and on the background mechanisms.
We need to engage in this kind of research to help understand the tumor biology of the African breast cancer especially in the LBC subset. We have been doing some research into some of the subsets of breast cancer but not the lobular type
Lobular breast cancer there is no clear prognosis for now
the risk of local recurrence could be impact
To get specific answer to treat ILC
pooling trials to increase numbers and power of results
Most times Trials does not enroll ILC
It will be difficult to run trials exclusively on ILC, therefore the best option to obtain knowledge is to include them in "general" trials but make sure teh trial is powered to answer the main question in the subset of ILC
because I think they are different diseases
because this setting patient need a different tratment/follow up
it is a dictinct group which was not addressed in such way before

It just have different behavior and most probably will need different treatment
Because it would be interesting and useful to have a deeper knowledge about ILC
Research is the way of development.
to increase the vailidity and accurancy
Due to the diagnostic difficulty of ILC
Because it is a different disease most of the time
That how we learn and redefine our guidelines
Their clinical behavior is distinct from ductal. results may be different
Relevant - need to know based on the smaller volume of these patients and the opportunity to improve outcomes
different molecular and biological behavior of the tumor
To see if there is a difference in these patients
Especially given often differential response to Neoadjuvant chemo in the absence of head to head comparison with endocrine
This is much needed information
To lead to some more plausible evidence base for ILC
different management
I believe recent evidence suggests that we should be treating ILC as a different disease entity.
ILC is biologically different from IDC and does not respond to treatment in the same way
screening and diagnosis new treatments ways to improve existing treatments
Because we need more information on how to best tailor the treatment to these patients.
Useful info, may behave differently
differences in detection ability
Different histology maybe equivalent to a new etiology, so it makes no sense to don’t do it.
Important differences
Imaging trials me demonstrate specific benefit for patients with lobular breast cancer as compared to breast cancer in general
We need to identify treatment outcome, recurrence, survival for ILC specifically, knowing they can behave and respond quite differently than IDC.
Different outcomes
ILC is common, so we need to establish treatments for lobular.

Understand for better management
If it was thought to be relevant
For reconstruction
look to see if outcomes differ
sensitivity to treatment for lobular type breast cancer
Because lobular carcinoma is rare, we may first find trends in lobular carcinoma and differences from IDC based on subset analysis.
10% of invasive cancers and therefore significant subgroup
The ILC and IDC have different molecular profiles and we need to do include subtyping in the design of future clinical trials aimed to test the efficacy of novel targeted treatments
different biology
Depends on studies' objectives.
ILC biology is different from IDC. Thus, it makes sens to explore other therapeutic options
Get more information about ILC
Different biology and thus different response to therapy
unique characteristics
Same answer as before.
neoadjuvent treatment
It is reasonable to define the clinical practice.
Considering the different biological and pathological findings in ILC, I expect that some of the findings would be different
Potential differences in outcome
different molecular profile, different treatment results
Refinement of clinical management (decisions on adjuvant CT, follow-up, targeted treatments enriched in ILC)
To help with the development of properly reproducible diagnostic criteria for ILC
LBC behave different. Resistance and late relapses.
if deemed appropriate on the biological rationale of the trial setting; to build solid evidence for differential effect of treatments in ILC vs IDC NOS.
vide infra
it is a different type of cancer with other molecular characteristics
Because this is the only way that we can decipher clinical important differences between ILC and other histological subtypes

Both the genomic pattern and the molecular biology of invasive lobular carcinoma are specific. Need to find the molecular therapeutic target to ILC.

I think the traditional breast cancer treatment with chemo is not useful for ILCand therefore should be avoided

Its a unique histology with different clinical pathological characteristics and patterns of recurrence

To improve outcomes for patients with lobular breast cancer

It’s an area of need and the data would be valuable

Outcomes May be different

If i could enroll enough lobular patients and use non-Recist criteria for evaluation this would be incredibly important data.

IDC and ILC are very different in the oncogenic drivers and their dependence on estrogen signalling

This is the most critical piece missing in allour hormone receptor positive studies. In the future, if the context is relevant for ILC, we should power the study to include certain number of ILC pts to be able to do subset analysis. This is the only we will be providing appropriate therapy to these patients.

Different characteristics of lobular cancer from ductal histology

The only way to learn about the specific relationship between lobular and response to therapy is to power the trial to answer this specific question. Otherwise, underpowered to measure this effect. then can't learn.

to improve ILC patients treatment

to determine if there is better mechanistic based therapy

Because histopathology can guide the diagnosis and treatment.

Sample size issues

In order to ascertain if the treatment intervention has more/less/same therapeutic value in ILC as IDC , so don't just keep treating ILC the same way as IDC

Clinical trials usually involve IDC. Lobular are excluded in many of them

The targeted treatment is necessary for ILC

It seems to be worse

lobular cancers have different metastatic spread and poorer survival

See answer above.

Increase in numbers to achieve robots statistics and to check treatment options from different hospitals and countries

The biology of lobular carcinoma is very different than ducal, nst

outcome might be different for ILC than non-ILC

I am involved with ISPY2 and we are classifying benefit of chemotherapy based on molecular and phenotypic subtupes. Would be good to evaluate lobular genomic (MammaPrint) high risk patients as an independent factor.

Different metastatic behavior

To demonstrate whether there is impact based upon subtype

To clearly assess different sensitivity to treatment in trial

To study this subset separately.

Different clinical, pathology and molecular features

ILC is still understudied and a lot of the standard treatments/predictive tools/GEPs/... that we use are not validated for ILC

Need clarification on different behaviours

There may be unique outcomes for ILC

Even within the same intrinsic subtype, the frequency of mutated genes are different between different histotypes. Thus precision oncology might play a role on developing unique treatment for lobular carcinoma.

n/a

ILC treatment should be as evidence-based as IDC.

targeted therapy in future

it is a different biology and we need to understand more about lobular carcinoma

increasing incidence and emerging data on disparate outcomes

Lobular cancer has different clinical behavior, genomic anomalies and sensitivity to existing agents.

To ensure there is no differential effect

depends on the type f trial, eg the role of specific -neo- adjuvant chemotherapy, or anti oestrogen treatments

Ideally yes. The challenge is to have a sufficient sample size for such a trial.

because of differences in therapy, prognosis and genetic risks

It is the only way to identify significant differences in response to therapy and outcome

This is a great area of need in clinical breast cancer research.

Would be great to do in some of the large adjuvant trials

because now I do'nt have a lot of experience with it, it could me interesting to learn more about it.

As long as you have a strong scientific justification, I am willing to adjust the design.

Most trials are underpowered for a subset analysis of ILC. Unfortunately oncologist think of breast cancer only in terms of receptor expression (triple negative, Her2+, etc) and pathologists go along

with that. This is an oversimplification that prevents us from doing doing proper studies on ILC. The situation is even worse for other subtypes.

I think there are biologic differences in lobular breast cancer that make this worth considering.

only if a different outcome is expected

To identify specific patterns of response/outcome in patients with ILac.

Sample size might be an obstacle

different disease, different response, different outcomes expected

will not advance treatment any other way

to help better understand this phenotype

It is a distinctly unique type of cancer with different patterns of clinical spread, response to therapy, and long term course. It is also a significant portion of breast cancer that deserves to be split out.

ILC is a molecularly distinct disease from IDC and may respond differently to study drug and treatment combinations. In HR+ breast cancer studies, stratification by histological subtype should be considered to ensure adequate representation of ILC (at least 10-15% of all included patients)

Differing underlying biology

I am convinced in the difference in biology between ILC and IDC. The vast majority of patients with HR+/HER2- BC in clinical trials have IDC histology. Conclusions might not apply mainly in chemo and endocrine therapy trials.

Chemo does not work

There are still a lot of questions on differences in biological behaviour and best treatment between NST and ILC. Results are a bit conflicting in literature

It’s important. Due to the lower numbers of ILC it is difficult to power for this

To achieve confirmatory results rather than retrospective subgroup results

ILC evolution differs depending on subtype

I have performed subgroup analyses for ILC in a formal prospective retrospective study and I consider prospective randomized trials with a comprehensive translational focus as the next important step.

Please see my prior reply

It would depend on the context and drug(s) used

ILC has different subtypes, each showing diferent repector profiles and prognosis.

Classical ILC may not be sensitive to chemotherapy, but may be sensitive to endocrine related therapy.

It would be interesting to have more strong data regarding this aspect

potential for differential efficacy by histology

The differences reported to date between ductal and lobular cancers are mostly due to higher proportion of high-grade tumors in ductal and low/intermediate grade tumor in lobular category. One should compare lobular and ductal matched for grade and receptors.
in the absence of high quality prospective data , the question if different treatment strategies are required will never be answered convincingly
I would like to know more about the difference in sensitivity to endocrine therapy. However, if the number of enrolled patients is small, the results of subgroup analysis may be difficult to interpret.
different clinical behavior
Need to improve knowledge about this type of cancer
- need to learn more about differences between lobular and ductal to have more effective treatments
It's a different beast akin to HER2 positive or TNBC
ILC could have a different response than IDC.
Because of the different biology as compared to nst
since it is a minority of BC patients practical issues and economic issues may make allowing for a 20%
There may differences in response.
Different responsiveness to systemic treatment
It's important to treat them

Supplementary Data 1. Names of survey participants

A Phillips
Abby Brody
Adam Brufsky, MD, PhD
Aditya Manna
Adrian Harris
Adrian Lee
Adriana van Beelen
Aimee Hare
Alana Welm
Alberto Porcu
Alexandre de Nonneville
Ali
Aline Goncalves
Alison Fox
Alison Penny
Allen Pannell
Almocera, Emelisa
Alua Jones
Alyse Siegel Chin
Amanda
Amanda Clark
Amanda Kasahara
Amcp
Amir Bahreini
Amit Agrawal
Amudha Saranathan
Ana Tecic Vuger
Andi Cani
Andrea moreno
Andrew L. Smith, PhD
Andrew Nelson
Angela Alexander
Angela Santry
Angela Swann
Angela Waweru
Anita
Ann Marie Rikenberg
Ann Perry
Ann Van Haney
Anna Churikova
Anna cariello
Anna, Mercanti
Annalisa La Cesa
Anne Beeckman
Anne Marie Mercurio
Anne Roche

Anne-Vibeke Laenkholm
Annette Lebeau
Anneza Yiallourou
Ariella Hanker
Ashley Bickford
Ashraf, Selim
Ayooye Samuel, Ajayi
Ayse Karaveli
BENUSIGLIO, Patrick
Barb DeMarco
Barbara Conway
Barbara Jacobsen
Barbara Neillsen
Barbara Radecka
Beate Kalstad Hernes
Bela Mrinakova
Belen Merck MD
Belinda Curpen
Belinda van den Berg
Ben Park
Bering, margaret
Bernadette AM Heemskerk
Bernadette Heemskerk-Ge
Beth Pelzer
Bette Weaver
Beverly Durham
Bhuvaneswari Ramaswamy
Blaise
Blessing, Zambuko
Bonnie Fleck
Bonnie Weck
Borbala Szekely MD, PhD
Brian Dubin
Bridget Mellett
COrissa Venettacci
CRISTINA NASURDI
Cahill Therese
Carlos Gallina
Carlos h dos anjos
Carol A Sartorius, PhD
Carol Dale Duby
Carol Deom
Carol Holmes
Carol Lanphear-Cook
Carol, Lange
Carole Troy
Caroline Keane
Carrie Anderson
Carrie Felice

Carrie M Brooks-Joiner
Catherine Loftus
Cathrin Brisken
Cathy Hirsch
Celena Latham
Chandler S Cortina, MD
Charmaine Macedo
Chen, Yu-Chih
Chi-Chen Hong
Chikako Honda
Chinar
Christel Fontaine
Christian D
Christina Addison
Christina C. Westhoff
Christine Desmedt
Christine Durlak
Christine Hodgdon
Christine Richardson
Christine Rosenberg
Christine Taylor
Claire Foden
Claire Kirby
Claire Turner
Clara Mulligan
Clare Cowhig
Claudia
Colleen Packer
Connie Hersbach
Constantine Dimitrakaki
Crissy
Cristiano Resende
Cristiano Souza
Cristina Buendia
Cyndi Troutman
Cynthia Doll
Cynthia Jorrie
Cynthia Moran
Cynthia Mynatt
Cynthia Wilcox
D. Joseph Jerry
DENISSE BRETEL
Dagmar Keita-DestrÃ©e
Dale Semler
Damir Vareslija
Dana Tonkin
Daniel Argolo
Daniel Hochbaum
Daniel Zingg

Daniele
Darlaine Honey
Darran O'connor
David N. Boone
Davut Can Guner
Dawn Cooper
Dawn Mooney
Deborah Shaw
Deborah Tibbs
Deborah Zanish
Dee Loughran
Deirdra Pickering
Denis Collins
Denis Larsimont
Denise Quayle
Dennis Sgroi
Denys Hannelore
Desaree Welch
Diane Deri
Diane Grandinetti
Diane Mapes
Dianne Clark
Dianne MacPherson
Djamila Fitzgerald
Donna
Donna Booth
Donna Charlevoix
Donna Cox
Donna Enz
Donna Kelly
Donna Kuljish
Donna Russell
Dorraya El-Ashry
Dr Flavia Sarquis
Dr. Beatrice Wiafe-Adda
Dr. Dimple Patel
Edoardo Isnaldi
Eduard-Alexandru Bonci
Eileen Strong
Elena Artamonova
Elia Mario Biganzoli
Eliana C. Martinez MD,
Elinor Sawyer
Elisa Agostinetto
ElisÃ©e Hategekimana
Elizabeth Repasky
Ellen Mandelbaum
Ellen Verschuur
Ellen Warner

Elsie Spry
Elspeth Ward
Elvira Bierdel-Willkomm
Elzbieta Senkus
Emi Noguchi
Emiel Rutgers
Emily Latacz
Emma Amos
Emma Ireland
Emma cornick
Erica Hobold
Erika, Klein
Eriko Narusawa
Erin Dotson
Eskil Fluge
Ethan Sokol
Etienne BRAIN
Eva Kuhn
Evandro de Azambuja
Evelyn Bordeaux
Evelyn Ruelas Bond
Ewa Folta-Stogniew
FRANCESCA, POGGIO
Fangyuan, Chen
Fatemeh Derakhshan
Fiona Duke
Fletcher, Jean
Flora migyanka
Flores, Aurora
FranÃ§ois Richard
G. Thomas Budd
Gabe Sonke
Gabriel Hortobagyi
Gabriele Zoppoli
Gary Ulaner
Gay Fright
Gaylene Hosking
Geoffrey Lindeman
George SFLOMOS
Geraldine Lavin
Gilberto Amorim
Ginny Mason
Giovanni Corso
Gitte Joergensen
Giuseppe Curigliano
Gloria Nicholls
Gopdjim LAURIANE
Gustavo Jankilevich
Gustavo Werutsky

Gwen Manchion
GÃ¡bor CSERNI
HERBERT KIM LYERLY
Hana Faistova
Hanna DillekÃ¥s
Hannah Ayettey
Harieta Garofide
Harikrishna Nakshatri
Harmanne Menkveld
Hayley Butler
Heather Hay Charron
Heather Roe-Lehman
Helen Gough
Helle Skjerven
HeloÃsa
Hilary Holden
Hilary Kepa
Hiroji Iwata
Huang, Yi
Ida Paris
JANE RILEE
Jacqui Taylor
Jamie Aochi
Jamila Simpson
Jane Bayani
Jane Lloyd
Jane Martin
Jane Peterson
Janet Briars
Janet Edwards
Janet Spitzer, MD
Janice Axelrod
Janice Bartley
Janina
Janine wozencroft
Jankowitz, Rachel
Jasmin Cooped
Jasmina NedoviÄ‡
Jason Carroll
Jean Douglas-Laird
Jean Murdoch
Jean Sershen
Jeananne Davis
Jeanie Ortis
Jeanine Nichols
Jeeyeon Lee
Jenise Jensen
Jenni R Harris
Jennie Hunt

Jennifer
Jennifer Dominick
Jennifer Halstead
Jennifer Harris
Jennifer Roger
Jennifer, McLin
Jennifer, Xavier
Jenny Burke
Jenny Katharina Wagner
Jens-Uwe Blohmer
Jessica Ralston
Jing Wang
Jo Browne
Jo wright
Joan E Ford
Joan Sullivan
Joanna Lamon
Joanne Louw
John Skoko
Jorge Reis-Filho
Jorge, Leal
Jos Jonkers
Jose Bazan
JosÃ© Palacios
Joyce Slingerland
Joyce Thorpe
Judith Rissia
Judith williams
Judy McDevitt
Julia Houthuijzen
Julia Levine
Julie Moore
Julie Swanson
Julie, Ostrander
Junichiro Watanabe
Justin Wells
Justine Neal
Kara
Karelia Wilkinson
Karen DiVito
Karen Van Baelen
Karez Namiq
Karin Kirkpatrick
Karolina Larsson
Kath Bray
Katheke Mbithi
Katherine Clifton
Kathleen Bordick
Kathleen faloon

Kathryn Krantz
Kathy DeHart
Kathy Hensley
Katja Kettler
Kay McKeon
Kazuaki Takabe
Kei Sugawara
Kelly McCann
Kelly Willner
Kerensa Dâ€™Arcy
Kerri Trail
Kevin Punie
Khadija Ring
Kim Graham
Kirstin Spencer
Kirsty McGhie
Kirtika Patel
Klara Julsing
Kristel Running
Kristie invie
Kristin L. Wohlschlagel
Kristine Blake
LESLIE WIZAN
Laura
Laura Czar
Laura Orgiano
Laura Savariau
Laura Trinidad Deocon
Laura mulholland fenton
Laura, Testa
Lauren Simpson
Laurie Hutcheson
Lavinia P. Middleton MD
Leanne Fryer
Leart Berdica
Leigh Pate
Leonor Matos
Lesley Beland
Libia Patricia
Liliane in t Veld
Lin, Chen
Linda Fuller
Lisa Carey
Lisa Costin
Lisa Emms
Lisa Hunter
Lisa Mastropieri
Lisa Matwick
Lisa Sobieski

Lori Carlson
Lori Petitti
Lorna McHattie
Lorraine Fraser
Louise Blanchard
Louise Woosey
Lounes Djerroudi
Lourdes Marx
Luc Dirix
Lucy Cafiero-Ahl
Lucy E Kohler
Luisa Carbognin
Lynley Clark
Lynne Wilkie
MARCELLA CARRILLO
MUKESH SHANTHILAL
Maartje Hooning
Madeline, Kreider
Mafalda Oliveira
Maggie Stresow
Mai Onishi
Makiko Kinjo
Mandy Gabriel
Marcia Falconer PhD
Margaret Buchanan
Margaret Troje-Meade
Margarita Figueroa
Maria
Maria Cristina Figueroa
Maria Cross
Maria P. Foschini
Marian Vanhoeij
Marianne Buekenhoudt
Mariela Vasileva-Slavev
Marilyn McWilliams
Marina Borges
Marion Maetens
Martin Blohmer
Mary
Mary Brennan
Mary Johnson
Mary McClurg
Mary McLaughlin
Mary O'Connor
Mary Serrilli
Mary Toner
Mary Weaver
Mary dominiecki
Mary morgin

MaryAnne Molter
Matthew Covington, MD
Matthew Sikora
Matthias Christgen
Maxim De Schepper
MaÅ‚gorzata Zauliczna
Megan-Claire Chase
Melany Chrash, MSN, APN
Melinda Wilcox
Melissa Southey
Meredith Wahlers
Merete Bae Flatin
Michael Gnant
Michaela Davis
Michele Atlan
Michele L Tremblay
Michelle Claud-Clemente
Michelle Porterfield
MieChen
Mieke Raap
Min Sun
Mina Youssef
Mireille Hogger
Missy Van Lokeren
Mitsuo Terada
Mohammed Ezzi
Monica Lis Casalnuovo
Monica Suarez Korsnes
Monique Arkesteijn
Monique Luchies
Mothaffar Rimawi
Mueller, Sophie
Mustafa Yilmaz
N'DA MARCELIN HOMIAN
Nancy Adams
Nancy Hara
Nancy Howell
Nancy Kershaw
Nancy Kiser
Nancy Ryan
Nancy Sobel
Nancy Wall
Nancy, Riess
Naoto Ueno
Natalie ter Hoeve
Nicole Dearing
Nicole Fuller
Nicoleta Antone
Nikolaos, Skouteris

Nina-Marie Opene
Nogueira Rodrigues, A
Norran H Said
Nunes, Dyego
OddbjÃ¸rn Straume
Oesterreich, Steffi
Oleksandr Berzoy
Olivia McGinn
Osama Shiraz, Shah
Pablo Guzman
Paige Olmo
Pam Taylor
Pamela Clarke
Pamela Dyer
Pamela Meyers
Paola, poletti
Patrha Roy
Patrice Kirchoff
Patricia Bates
Patricia Kaminski
Patrick Neven
Patrick, Derksen
Patti Watson
Patty Jackson
Paul J van Diest
Paula Brooks
Paulina Edith Gonzslez
Payal Abrol-Virji
Peg Mahoney
Peggy
Peggy Workman
Peter Lucas
Philippe Aftimos
Phina casey
Pieter Westenend
Prital Patel
Rachael Edmond
Rachael Natrajan
Rachele Caputo
Rafael LÃ³pez
Rafael Villanueva
Raquel Nunes
Ravi Patnaik
Rebecc Shatsky
Rebecca Birks
Rebecca Elliott
Rebecca Riggins
Regis Paulinelli
Renata Cangussu

Renata Chromczsk
Renee Flaherty
Rhonda Skeete
Rhonda Watson
Richard Simcock
Rika Kizawa
Rita, Canas-Marques
Ritse Mann
Robert Wesolowski
Roberta Prioleau
Robin Cohl
Robin Salter
Rochelle Strenger
Rohit Bhargava
Ron Bose
Ros Sharkey
Rosalia, Cordo Russo
Rose Arendarczyk
Rosemary Walsh
Rowanna Mc Guigan
Ruth Warden
Ruth, Keri
S Newby
SILVANA JULIA ROMPATO
SInead McDonagh
Sabine Linn
Sabine S. Spitz
Sandi Saltiel
Sandra Fuller
Sandra Swain
Sandro Cavallero
Sankari Nagarajan
Sara Johnston
Sarah Feeley
Sarah Nyante
Sarah Pinder
Sarah payne
Sasagu Kurozumi
Sean Egan
See Featherstone
Shana Rodgers
Shane Richmond
Shannon Donovan
Shannon Gryszkiewicz
Shannon Puhalla
Shari Beck
Shauna Golightly
Shauna Nyrose
Sherry Prinsen

Sheryl Kasper
Shoko Emily Abe
Silvina Bortnik
Simon Johnston
Sinead Carey
Siobhan Cogley
SiobhÃ¡n Freeney
SiÃ¢n Mollart
Slaney Mullen
Stella Mc
Stephani Christensen
Stephanie Fricas
Steven Cai
Su Prentice
Sue Beuning
Sue Corralz
Sunati Sahoo
Suryakanta Acharya
Susan Barton
Susan Gillies
Susan Jane Miller
Susan M Cummins
Susan MacDonald
Susan Sleeman
Susan christian
Susanne Dieroff Hay
Susanne Sprague
Susanne Topp
Suzanne Leigh
Suzanne Raffellini
Suzanne Szul
Szasz, A. Marcell
Szilvia Varga
Takahiro Kogawa
Tamara Milagre
Tammy McLean
Tammy Strickland
Tammy Villeneuve
Tatiana Vidaurre
Tengku Ahmad Damitri Al
Teresa Perra
Terri Coutee
Theresa Guise
Therese Murphy
Tian, Du
Tiffany Converse
Tina Ashton
Tomohiro Chiba
Tomomi Fujisawa

Tone
Topp
Tracy Cushing
Tracy Needham
Trish Hayes
Ulrik Narbe
VEGA IRANZO (ORCID ID:
VINCENT SALOMON ANne
Valente, Stephanie
Valerie Gaynor
Valerie Poe
Valerie Robson
Vanessa Prohl
Vanessa Sammut
Vera Cherepinsky
Vered Stearns
Veronique Debien
Veronique Dieras
Vicente, Marco
Vicki Bishop
Vickie McMurray
Vickie-Lee Wall
Vicky
Victor Kok
Vikki Groom
Vincent DeGennaro
Vincent Massard
Vivien James
Wai-Choo Finch
Wendy Bourke
William Muller Ph.D.
William, Yang
Xiao Huang
Xiaoxian Li
YM, Ho
Yahaira MelÃ©ndez
Yasemin, Kayadibi
Yates, Megan
Yayoi Adachi
Yi Li
Yongfei Li
Yousef Sharaiha
Yukio Koibuchi
Zsolt, Horvath
chris lord
claudia d'alessandro
hans wildiers
joanna stricker
lorenzo gianni

luciana landeiro
marc lippman
paul jibrin
sheryl, kasper
yong pan

Supplementary Data 2. Countries of survey participants

Afghanistan
Albania
Argentina
Australia
Austria
Belgium
Botswana
Brazil
Brunei Darussalam
Bulgaria
Cameroon
Canada
Chile
China
Côte d'Ivoire
Croatia
Cyprus
Czech Republic
Denmark
Dominican Republic
Egypt
El Salvador
Finland
France
Germany
Ghana
Greece
Haiti
Hungary
India
Iran
Iraq
Ireland
Israel
Italy
Japan
Kenya
Kuwait
Malaysia
Malta
Mexico
Netherlands
New Zealand
Nigeria
Norway
Peru

Philippines
Poland
Portugal
Romania
Russian Federation
Rwanda
Saudi Arabia
Serbia
Slovakia
Slovenia
South Africa
South Korea
Spain
Sweden
Switzerland
Thailand
Turkey
Ukraine
United Kingdom of Great Britain and Northern Ireland
United States of America

Supplementary Data 3. Funders for ILC research of survey participants (laboratory-based researchers)

Academy of Medical Sciences (UK)
American Cancer Society
Barcelona, Spain (BCN)
Breast Cancer Now
Breast Cancer Research Foundation (BCRF)
Breaskthrough Breast Cancer Research Foundation
Cancer Australia
Cancer League Colorado
Cancer Research UK
CCSRI (Canadian Cancer Society Research Institute)
CIHR (Canadian Institutes of Health Research)
CRUK Studentship awards
CPRIT
Department of Defense
Dutch Cancer Society (KWF)
Dutch Research Council (NWO)
Dynami Foundation
EU COST Action Lobsterpot
European Union
Fondation Luxembourg
Genentech ROCHE
German Cancer Aid (Deutsche Krebshilfe)
Golfer against Cancer
Hillman Fellowship
Institut Curie
Internal hospital grant
Irish Cancer Society
Irish Research Council
Italian Ministry of Health
ITALIAN ASSOCIATION Research against Cancer (AIRC)
LBCA
Magee Women's Research Institute & Foundation
METAvivor
Ministry of Science
National Health and Medical Research Council (Australia)
NCI (National Cancer Institute)
NIH (National Institutes of Health)
NWO (Dutch organisation for scientific research, veni grant)
Oncode Institute
Pathological Society (UK)
Pink Ribbon
Private sources
Royal Dutch Cancer Society
Ruban Rose Ligue contre le cancer
SAMAS EUSOBI RSNA
Shear Family Foundation

Spanish Government
Susan G Komen
Swiss Cancer League - Oncosuisse
The Scientific and Technological Research Council of Turkey
UZ/KU Leuven local funding COST action (ELBCC)
Umberto Veronesi Foundation
Unrestricted grant from Roche (for a neoadjuvant study in early ILC - ROSALINE)
University funding
University of Colorado Comprehensive Cancer Center Tumor Host Interactions Program
University of Nottingham (UK)
Wellcome Trust (through a clinical PhD studentship)
Worldwide Cancer Research

Supplementary Data 4. Advocacy groups, support groups, and other foundations of survey participants (patients)

Advocates for Breast Cancer in South Africa
After Breast Cancer Diagnosis (ABCD)
Agder Brystkreftforening
Alamo Breast Cancer Foundation
American Cancer Society (ACS)
Annie Appleseed Project
American Cancer Society Reach to Recovery program
Arc Cancer Support
AreYouDense.org
Association for Cooperation and Transformation (ACT) UK
Being Dense
Beyond the Pink Moon
Bezzy
B-Lief
Borstkanker Vereniging Nederland
Bosom Pals
BPAQ
Breast Cancer Action
Breast Cancer Care & Research Fund
Breast Cancer Coalition of Rochester
Breast Cancer Education Association
Breast Cancer Integrative
Breast Cancer Network Australia (BCNA)
Breast Cancer Now
Breast Cancer Research Advocacy Network (bcRAN)
Breast Cancer Research & Awareness Unit
Breast Cancer Research Foundation
Breast Cancer Support
Breast cancer tumor board
BreastCancer.org
Breast Care International
Breast Density Matters
Breast Friends
Breast Tissue Bank Brisbane
BreastCancerAdvocacy.org
Breastlink
Briah Fund
Bridge of Health
Brystkraft Facebook group
Building Resilience in Breast Cancer (BRiC)
BVN
California Breast Cancer Research Program (CBCRP)
Canadian Breast Cancer Network
Canadian Cancer Survivor Network
Cancer Association
Cancer Connections

CBC
CCO
Chinese Society of Clinical Oncology (CSCO)
Connect Metastatic Breast Cancer online group
Conquer Cancer, the ASCO Foundation
Dense Breast Info (DenseBreast-info.org)
Dense Breasts Canada
Diep Reconstruction
Egyptian National Breast Cancer Screening Program
Egyptian Society of Women's Health
Europa Donna
European Cancer Patient Coalition (ECPC)
European Lobular Breast Cancer Consortium
European Patients' Academy on Therapeutic Innovation (EUPATI) Austria
European Reference Network for Genetic Tumor Risk Syndromes (ERN Genturis)
Facing Our Risk of Cancer Empowered (FORCE)
Femama
Foundation for Breast Cancer Center (FfBCC)
Geneticancer
Ghana Non Communicable Diseases (NCD) Alliance
Global Initiative for Breast Cancer Awareness Egypt
Groupe de Support Contre le Cancer
Grupo Argentino de Investigación Clínica en Oncología (GAICO)
Grupo Brasileiro de Estudos do Câncer de Mama
Guiding Researchers and Advocates to Scientific Partnerships (GRASP)
Healing Odyssey
Hopkins INSPIRE Research Advocacy
Hunter Women's Breast Cancer support group
ICanServe Foundation, Inc.
ILC
ILCA
Imerman Angels
Inflammatory Breast Cancer Research Foundation
Innovation for Perfection in Health
Instituto Lado a Lado
Instituto Nacional de Enfermedades Neoplásicas
Invasive Lobular Breast Cancer Association
Irish Cancer Society
Kenya Cancer Association
Komen Advocates in Science
Lega Italiana per la Lotta contro i Tumori
Living Beyond Breast Cancer (LBBC)
LLBC
Lobular Alliance UK
Lobular Breast Cancer
Lobular Breast Cancer Alliance (LBCA)
Lobular Breast Cancer UK
Lobular Carcinoma In Situ (LCIS) Alliance
Local support groups

Lymphoedema Queensland
Male Breast Cancer Coalition
Mama Oncoclinicas
mamazone e.V.
Marie Keating Positive Living Group
MBCBrainMets.org
Met Up UK
Metaplastic Breast Cancer Global Alliance
Metaplastic Breast Cancer Support and Information
Metastatic Breast Cancer Alliance (MBCA)
METAvivor
Metro South Health
METS (support group)
Minimal Access Surgery (MAS) Rural Initiative
Morgan Welch Inflammatory Breast Cancer (IBC) Program
Mt. Sinai Survivors
Mulika Saratani
Mutant Strong
Narikeldaha Prayas
National Breast Cancer Coalition (NBCC)
National Breast Cancer Foundation
National Breast Cancer Group
National Breast Cancer Screening Program
National Cancer Institute (NCI)
Nationally Consistent Collection of Data (NCCD)
New Hampshire Breast Cancer Coalition
Norges Brystkreftforening
Norwegian Breast Cancer Group (NCBG)
Norwegian Cancer Society
Oncoguia
Patient-Centered Dosing Initiative (PCDI)
PAY-W Clinic India
Peace and Love Survivors Association
Pennsylvania Breast Cancer Coalition
Peruvian Breast Cancer Club
Pink & MetaPink Stars
Pink Bolero
Pink Ribbon Foundation
Prince Philip Hospital
Rays of Hope
Reach to Recovery International
Roche IHC Group Nigeria
Rosa e Amor
Różowy Motyl
Sabah Kinabalu Pink Ribbon
Sea of Change Choir
Sensitisation, Screening, and Diagnosis
SHARE Cancer Support
Sharsheret

Shibukawa Medical Center
Society for Cellular Pathology of Nigeria
Society of Breast Imaging
SOLTI
Specialized Program of Research Excellence (SPORE) at Lineberger Cancer Center
Stage 4 Deserves More
Support for People with Metastatic Lobular Breast Cancer (Facebook)
Susan G Komen
Surviving Breast Cancer
Swedish Breast Cancer Association
Taiwan Breast Cancer Alliance
Tamoxifen support group
Team Judy Fund for Metastatic Breast Cancer Research (Team Pedicure) The Alliance of Oncological Patient Organisations
The Answer to Cancer
The Dragon Flies Breast Cancer Survivor Group Newmarket
The Inflammatory Breast Cancer (IBC) Network Foundation
The Nairobi Hospital Cancer Group
The Pink Journey Foundation
The Rose Houston
Translational Breast Cancer Research Consortium (TBCRC)
Union of International Cancer Control
Unite for HER
University of California San Francisco Lobular Breast Cancer Research Program
University of Texas Southwestern Research Advocacy Team
With You Japan
Young Survival Coalition (YSC)

Supplementary Data 5. Questions related to patient-physician communication

Country	Q46(Did your physician/health provider explain the unique features of ILC including how it presents differently from ductal?)			
	No	Yes	Don't know/ can't remember	Total
US	191	201	17	409
	46.7	49.14	4.16	
GRB	92	56	8	156
	58.97	35.9	5.13	
IRL	49	30	5	84
	58.33	35.71	5.95	
NTH	19	14	1	34
	55.88	41.18	2.94	
OTH	74	43	9	126
	58.73	34.13	7.14	
Total	425	344	40	809

*Chi-Square test p-value 0.0362

Country	Q47(Did your care team discuss personalized therapy with you based upon your histologic diagnosis (i.e. IDC vs ILC)?)				
	No	Yes	Don't know/ can't remember	I was not offered personalized therapy because my physician explained that the treatment is no different for ductal than for lobular	Total
US	142	103	21	144	410
	34.63	25.12	5.12	35.12	
GRB	82	26	14	34	156
	52.56	16.67	8.97	21.79	
IRL	46	12	14	12	84
	54.76	14.29	16.67	14.29	
NTH	15	4	2	13	34
	44.12	11.76	5.88	38.24	
OTH	61	23	4	38	126
	48.41	18.25	3.17	30.16	
Total	346	168	55	241	810

*Chi-Square test p-value <0.0001

Country	Q49(Did your physician/ health provider explain how to monitor yourself for local or distant recurrence in the future*			
	No	Yes	Don't know/ can't remember	Total
US	150	112	25	287
	52.26	39.02	8.71	
GRB	68	35	9	112
	60.71	31.25	8.04	
IRL	38	16	3	57
	66.67	28.07	5.26	
NTH	15	8	2	25
	60	32	8	
OTH	49	27	5	81
	60.49	33.33	6.17	
Total	320	198	44	562

*Chi-Square test p-value n.s.

** Only patients with stage I and II were asked

Country	Q50(Did your physician/ health provider explain that lobular cancer cells can metastasize to unique places?)			
	No	Yes	Don't know/ can't remember	Total
US	258	125	24	407
	63.39	30.71	5.9	
GRB	132	18	6	156
	84.62	11.54	3.85	
IRL	62	18	4	84
	73.81	21.43	4.76	
NTH	27	5	2	34
	79.41	14.71	5.88	
OTH	97	22	7	126
	76.98	17.46	5.56	
Total	576	188	43	807

*Chi-Square test p-value <0.0001

Country	Q51(Did your physician/ health provider explain what symptoms, including unusual symptoms, of recurrence you should report in the future?)			
	No	Yes	Don't know/ can't remember	Total
US	303	81	23	407
	74.45	19.9	5.65	
GRB	127	22	7	156
	81.41	14.1	4.49	
IRL	71	9	4	84
	84.52	10.71	4.76	
NTH	31	2	1	34
	91.18	5.88	2.94	
OTH	104	19	3	126
	82.54	15.08	2.38	
Total	636	133	38	807

*Chi-Square test p-value n.s.

Supplementary Data 6. GPT-4 summarization of ILC-related topics which patients wished they have had a chance to discuss with their physicians

Gpt-4 summarization to the initial 10 clusters ("Ambiguous" group (cluster 11) not included):

Cluster 1: ILC unique features

Patients with invasive lobular carcinoma (ILC) express a desire for more comprehensive discussions with their healthcare providers about the unique characteristics, behavior, and long-term recurrence risks of ILC, as well as the differences between ILC and other types of breast cancer, such as invasive ductal carcinoma (IDC).

Cluster 2: Surgery

Patients with invasive lobular carcinoma (ILC) express a desire for more comprehensive discussions with their physicians about the various surgical options, particularly the benefits and consequences of mastectomy versus lumpectomy, and the potential for recurrence post-mastectomy.

Cluster 3: Personalized treatment and mental care

Patients with invasive lobular carcinoma (ILC) express a desire for more personalized, nuanced treatment options, including alternative therapies and preventative measures, with a particular emphasis on understanding the potential complications and impacts of such treatments on overall health and well-being.

Cluster 4: Cancer detection and screening

The common topic among the responses is the challenge in detecting Invasive Lobular Carcinoma (ILC) using traditional imaging techniques, emphasizing the need for alternative screening methods such as MRI for more accurate diagnosis and monitoring.

Cluster 5: Recurrence

Patients with invasive lobular carcinoma (ILC) express a desire for more comprehensive information from their healthcare providers regarding the risk, signs, and monitoring strategies for potential cancer recurrence.

Cluster 6: Metastasis

The common topic among the responses is the desire for comprehensive information regarding the unique metastatic patterns of Invasive Lobular Carcinoma (ILC), including potential sites, associated symptoms, detection difficulties, and implications for prognosis and treatment.

Cluster 7: Influence of breast density

The common topic among these responses is the difficulty in detecting Invasive Lobular Carcinoma (ILC) in dense breasts using traditional mammography, leading to a need for alternative imaging techniques such as MRI and ultrasound, and the potential for recurrence in the contralateral breast.

Cluster 8: Chemotherapy

The common topic shared among the answers is the effectiveness and necessity of chemotherapy in the treatment of Invasive Lobular Carcinoma (ILC).

Cluster 9: Endocrine therapy

Patients with invasive lobular carcinoma (ILC) express a desire for more comprehensive discussions with their healthcare providers about the potential side effects of treatments, including hormone therapies such as aromatase inhibitors (AIs) and tamoxifen, as well as the long-term impacts of these treatments on their overall health and quality of life.

Cluster 10: Radiation therapy

The common topic among the responses is the need for comprehensive discussion and understanding of the potential benefits, risks, side effects, and long-term implications of radiation therapy in the treatment of Invasive Lobular Carcinoma (ILC).

Data File 7. Most critical and impactful ILC research questions

	Physicians	Lab-based Researchers	Patients	p-value
**Epidemiology and Risk Reduction**				
Therapy, Treatment Resistance and Disease Progression	43	50	60	<.0001
Identifying strategies to improve ILC screening/early detection	68	73	89	<.0001
Impact of obesity and lifestyle factors on risk of developing ILC and risk of relapse	38	38	50	0.0006
**Diagnosis (Imaging and Pathology)**				
Examining use of E-cadherin/p120 expression	52	55	74	<.0001
Improving diagnosis and understanding of mixed IDC/ILC	50	56	65	0.0002
Understanding the use of artificial intelligence	41	37	60	<.0001
Role of genomic predictors for ILC prognosis and prediction of therapeutic response	75	78	76	0.6822
Identifying strategies to improve ILC screening/early detection	73	73	90	<.0001
**Therapy, Treatment Resistance and Disease Progression**				
Identifying mechanisms of metastases	71	75	88	<.0001
Determining mechanisms of endocrine resistance in ILC	81	80	84	0.242
Identification of novel therapeutic targets and/or repurposing existing drugs for ILC	78	81	84	0.1775
Determining utility of immunotherapy in ILC	65	60	79	<.0001
Understanding value of liquid biopsies in patients with ILC	52	56	73	<.0001
Characterizing chemotherapy in ILC, and understanding differences to IDC	74	67	77	0.0034
Determining mechanisms of dormancy and risk for late relapse	70	73	87	<.0001
Developing and testing lifestyle interventions	34	32	48	<.0001
**Imaging**				
Optimizing current breast cancer screening modalities	62	60	83	<.0001
Identifying new and specific imaging tools for ILC	65	68	92	<.0001
Studying the importance of breast density	44	42	76	<.0001
Determining the utility of MRI	65	58	80	<.0001
**Local therapy of the Primary Tumor**				
Determining how to reduce the high positive margin rates in ILC	66	60	76	<.0001
Characterizing further whether breast conservation/radiation is as safe as mastectomy	52	50	63	0.0003
Determining whether radiotherapy can replace axillary surgery in ILC	57	52	61	0.039
Characterizing difference in post-mastectomy radiation between ER+ IDC and ER+ ILC	50	42	61	<.0001
**Basic/translational research question**				
Determining cell of origin for ILC	39	43	73	<.0001
Development of a centralized ILC data and tissue registry	56	70	77	<.0001
Developing and characterizing ILC models	47	70	71	<.0001

Characterizing differences in the tumor microenvironment between ILC and IDC	54	71	72	0.0001
Understanding of LCIS as a precursor ILC	43	46	60	<.0001
Characterizing subtypes of ILC (pleomorphic, mixed etc)	47	51	63	0.0001
Understanding of the unique etiology of ILC	46	49	66	<.0001

* Green: Highest rating within domain by stakeholder group
* Red: Highest 2 ratings by group across all domains

Suppl data File 8

Please indicate below other research questions that have high priority that we have not listed:

(in order of frequency of responses):

1 Genetic screening. Germline mutations. Familiar risks. 27
2 Awareness education 19
3 AI and SERDs (new drugs and duration) 13
4 Genomic predictors markers 12
5 Chemotherapy 10
6 Environment. Nutrition. Alternative approaches. 9
7 ctDNA. Liquid Biopsies. 9
8 Age (ie ILC in youndger or elderly patients) 8
9 PET 8
10 ILC variants 7
11 Dormancy and late recurrences 7
12 Pregnancy. Breast feeding. Contraception. HRT 5
13 Race. Disparities. 5
14 Metabolism. 4
15 Neoadjuvant treatment. 3
16 MRI 3
17 IO 2
18 Big data and AI 2
19 Gut microbiome 1
